# Supplementary material for: Allosteric regulation and crystallographic fragment screening of SARS-CoV-2 NSP15 endoribonuclease
Source: Nucleic Acids Res. 2023 Apr 28;51(10):5255–70. doi: 10.1093/nar/gkad314 (PMC10250223; doi:10.1093/nar/gkad314)
Supplement: gkad314_Supplemental_Files [file gkad314_supplemental_files.zip › Supplementary datasets.zip!/231/Refine_8/01-BUSTER/Cycle-5/LIST.html]

Refining

**BUSTER 2.X**: A suite of programs for macromolecular structure refinement by maximum-likelihood and Bayesian methods  
Copyright © 1992-2020 Global Phasing Ltd.  
All rights reserved.  
Authors: G. Bricogne, E. Blanc, M. Brandl, C. Flensburg, P. Keller, W. Paciorek, P. Roversi, O. Smart, C. Vonrhein, T. Womack  

Please reference use of the software as:

- BUSTER-TNT 2.X, Global Phasing Ltd, Sheraton House, Cambridge CB3 0AX, UK

Version: 2.10.3 for linux64-ifort made on May 20 2020 at 01:14:34  
Licenced to: Diamond Light Source, Scientific Software  

```
---


### Parsing of the BUSTER cardfile

TITLE : autoBUSTER refinement

   Cell information

   Symmetry information

 Using CCP4 X-ray form factors.

   Missing structure chemical composition

 Using ATOM block for chemical composition.


   Shell information

### Parsing of the TNT cardfile


---


### Reading observations file

 Number of reflexions in input:  85622

      Number of accepted reflexions:  81660

      Number of rejected reflexions:      0

   Preparing complete.mtz containing:      85561 reflexions


   Reflexions in test set:            4077

   Reflexions in working set:        77583


---


### 1. Generating initial model

 2.10 * Shannon sampling rate. NX NY NZ:   360  360  240


  Effective Shannon rates are:    2.50    2.50    2.26


   Allocated BUFFER of size:    119.312 Mb.

   Initial Geometry Information


 No missing atoms declared


   Summary of model volumes

 No missing atoms declared: no prior computed


 Computing bulk solvent model scattering


 NCSMASK PDB radius for solvent mask:     2.15 Angstrom

 running PDBSET (setting cell and spacegroup P1) ... done
 running NCSMASK [1] (creating mask, SMOOTH 0.921083) ... done
 running MAPDUMP (to check if reduction to unit cell is needed) ... done
 running MAPMASK (converting mask to map) ... done

   Generation of babslv by theta-filter

   Electron density in frg mask:  0.420 Å-1

 FOBS  put on rough absolute scale by Wilson plot :
           K        =  0.39257E+02
           B        =    -11.084

 

### Cycle 0001: MaxLik scaling and refinement


   MaxLik Scaling Cycle 0001

   Wilson plot

   Statistics in resolution bins just after scaling
```

|  |  |
| --- | --- |
|  | - Rxpct factors plot- Rxpct factors table- <Log-Lik Gain>- Ln Sigma A- Correlation Coefficients |

```
   <Log-Likelihood> of null-hypothesis

Structure Factor Amplitudes


   Average Figure of Merit

 Switching off refinement of overall temperature factor


 Added B =     0.0211 Å2 to all atoms in fragment


 Added B =     0.0211 Å2 to B_SOLV

```
gelly refinement module
 Authors (2004-2009) O. S. Smart, C. Flensburg, W. Paciorek, C. Vonrhein, 
                     M. Brandl, T. O. Womack and G. Bricogne
         (2010-2011) O. S. Smart, C. Flensburg, W. Paciorek, C. Vonrhein, 
                     T. O. Womack and G. Bricogne
         (2012-2014) O. S. Smart, C. Flensburg, W. Paciorek, C. Vonrhein, 
                     and G. Bricogne
         (2015-2020) G. Bricogne, C. Flensburg, W. Paciorek, C. Vonrhein
 Copyright © 2004-2020 Global Phasing Ltd. 
 All rights reserved.
 gelly module compilation date May 20 2020 01:11:20
 
 gelly uses routines from TNT geometry. References: 
     Tronrud, D. E., Ten Eyck, L. F., Matthews, B. W. (1987)
     "An Efficient General-Purpose Least-Squares Refinement Program 
     for Macromolecular Structures". Acta Crystallogr A, 43:489-501.
     Tronrud, D. E. (1997). "The TNT Refinement Package", 
     in Macromolecular Crystallography, Part B, Eds Charlie Carter 
     and Robert Sweet, Methods in Enzymology, 277:306-319.
 wildcard matching function by Julian Robichaux http://www.nsftools.com

 No -random_seed flag found. Random number stream initialised using:
2147483562
2147483398

 Control flags set to:  -autoncs -hydrogens_input_occ2zero \
                     -hydrogens_output_occ2heavy -screen \
                     100 -screen_sigma \
                     3.0 -keeppdb \
                     1000000 -taomonitor_verbose \
                     2 
 

 ############### Starting setup via TNT routines

 Setting up tnt routines including license check:

                   TNT Stereochemistry Program
 Version: 5.22.3.0
 Licence for Diamond Light Source, Scientific Software is valid
 
 Using TNT geometry routines to load the tnt control file: gelly.tnt
 Using TNT routines to load initial coordinates from file fragment.cor
 Expecting 6138 atoms

 Found     2195 "NOTE BUSTER_RESIDUE_ATOM_DATA" cards
 Have copied information for    6138 atoms from TNT to gelly's store 
 
 The TNT WEIGHT cards used by this run:
 -----WEIGHT       BOND     2.0
 -----WEIGHT       ANGLE    2.0
 -----WEIGHT       IMPROPER 0.0
 -----WEIGHT       TORSION  2.0
 -----WEIGHT       PSEUDO   0.0
 -----WEIGHT       TRIGONAL 2.0
 -----WEIGHT       PLANE    5.0
 -----WEIGHT       CONTACT  5.0
 -----WEIGHT       BCORREL  10.0
 -----WEIGHT       CHIRAL   5.0
 -----WEIGHT       NCS      50.0
 -----WEIGHT       IDEAL    4.0
 -----WEIGHT       RFACTOR  5.82
 
 Dictionary versions picked up from NOTE BUSTER_RESTRAINT_DICT cards as: 
 ------ protgeo_eh99.dat (V1.6.4.17) 20191001  STANDARD AMINO ACID DICTIONARY. BONDS AND ANGLES FROM ENGH AND HUBER EH99. OTHER VALUES BASED ON PREVIOUS TNT OR TAKEN FROM CCP4. INCLUDES HYDROGEN ATOMS.
 ------ exoticaa.dat (V1.3.4.15) 20200327  COLLECTION OF NON-STANDARD AMINO ACIDS, MAINLY EH91 WITHOUT IDEAL DISTANCE INFO
 ------ nuclgeo.dat (V1.13.4.8) 20190909
 ------ bcorrel.dat (V1.15.10.2) 20190616
 ------ contact.dat (V1.15.12.10) 20191121
 ------ idealdist_contact.dat (V1.3.4.12) 20191001  IDEAL-DISTANCE CONTACT TERM DATA AS USED IN PROLSQ. VALUES USED HERE ARE BASED ON DATA HARVESTING OF PDB AS OF 20190109.
 ------ restraints for LIG (Unknown) from cif dictionary POB0120.cif
 ------ restraints for CIT (CITRIC ACID) from cif dictionary CIT.cif; generated by GRADE_PDB_LIGAND 1.2.12 (October 4 2016) using MOGUL 1.7.2(RC1), CSD as538be, with quantum mechanics RM1 ((DatabaseID PDB-CIT))
 ------ assume.dat (V1.9.4.2) 20180705
 
 Have picked up spacegroup name for pdb output as 'P 63      '
 Setting up Chain/Residue arrays
 
 Have command line specification -autoncs*
     Have found       1 NCS relations
     Pairwise matching residue number and type between chains: 
                    A        B        S    
          A         .      100%       -   
          B                  .        -   
          S                           .   
       Table key--                                                       
          all   : two chains have 100% match and same number of residues 
         multi  : ditto and they contribute to a multi chain ncs cluster 
          ***%  : two chains have >80% matching residue number/type      
           -    : chains appear to be unrelated                          
      chain A     have identified ncs to     1 other chains. So will weight each autoncs by (       6.666/#)=      6.6660
      chain B     have identified ncs to     1 other chains. So will weight each autoncs by (       6.666/#)=      6.6660
      chain S     have identified ncs to     0 other chains. 
     For .Gelly cards produced see below - after pruning
 
 Setting up BUSTER_SET groups
 Have found    89 BUSTER_SET cards
 Now silently applying automatic/dictionary set definitions. For a verbose listing use '-verbose_set' flag.
 Applying user defined set definitions:
 =====NOTE BUSTER_SET ncsautoXcld = Water --> set 'ncsautoXcld' now has   581 atoms 
 =====NOTE BUSTER_SET ncsautoABset = Chain_A \ ncsautoXcld --> set 'ncsautoABset' now has  2785 atoms 
 Have defined    48 sets: 
      set:                 'All' has   6138 atoms in   1282 residues - automatically defined
      set:               'Empty' has      0 atoms in      0 residues - automatically defined
      set:            'ExoticAA' has      0 atoms in      0 residues - defined by dictionary file
      set:           'ExoticNUC' has      0 atoms in      0 residues - defined by dictionary file
      set:              'NotHET' has   5514 atoms in    698 residues - defined by dictionary file
      set:             'Chain_A' has   2785 atoms in    351 residues - automatically defined
      set:             'Chain_B' has   2772 atoms in    350 residues - automatically defined
      set:             'Chain_S' has    581 atoms in    581 residues - automatically defined
      set:               'Water' has    581 atoms in    581 residues - defined by dictionary file
      set:          'StdProtein' has   5514 atoms in    698 residues - defined by dictionary file
      set:             'Protein' has   5514 atoms in    698 residues - defined by dictionary file
      set:                'Back' has   2794 atoms in    698 residues - defined by dictionary file
      set:                'Side' has   2720 atoms in    650 residues - defined by dictionary file
      set:        'RnaCanonical' has      0 atoms in      0 residues - defined by dictionary file
      set:        'DnaCanonical' has      0 atoms in      0 residues - defined by dictionary file
      set:     'RnaDnaCanonical' has      0 atoms in      0 residues - defined by dictionary file
      set:     'DnaRnaCanonical' has      0 atoms in      0 residues - defined by dictionary file
      set:         'RnaExtended' has      0 atoms in      0 residues - defined by dictionary file
      set:         'DnaExtended' has      0 atoms in      0 residues - defined by dictionary file
      set:      'RnaDnaExtended' has      0 atoms in      0 residues - defined by dictionary file
      set:      'DnaRnaExtended' has      0 atoms in      0 residues - defined by dictionary file
      set:                 'Dna' has      0 atoms in      0 residues - defined by dictionary file
      set:                 'Rna' has      0 atoms in      0 residues - defined by dictionary file
      set:              'RnaDna' has      0 atoms in      0 residues - defined by dictionary file
      set:              'DnaRna' has      0 atoms in      0 residues - defined by dictionary file
      set:             'Nucleic' has      0 atoms in      0 residues - defined by dictionary file
      set:             'Polymer' has   5514 atoms in    698 residues - defined by dictionary file
      set: 'BufferWeichenberger' has     13 atoms in      1 residues - defined by dictionary file
      set:    'BufferAdditional' has      0 atoms in      0 residues - defined by dictionary file
      set:              'Buffer' has     13 atoms in      1 residues - defined by dictionary file
      set:         'MetalAlkali' has      0 atoms in      0 residues - defined by dictionary file
      set:  'MetalAlkalineEarth' has      0 atoms in      0 residues - defined by dictionary file
      set:     'MetalTransition' has      0 atoms in      0 residues - defined by dictionary file
      set: 'MetalPostTransition' has      0 atoms in      0 residues - defined by dictionary file
      set:    'MetalLanthanides' has      0 atoms in      0 residues - defined by dictionary file
      set:               'Metal' has      0 atoms in      0 residues - defined by dictionary file
      set:         'SugarAlphaD' has      0 atoms in      0 residues - defined by dictionary file
      set:         'SugarAlphaL' has      0 atoms in      0 residues - defined by dictionary file
      set:               'Sugar' has      0 atoms in      0 residues - defined by dictionary file
      set:               'Other' has     43 atoms in      3 residues - defined by dictionary file
      set:            'Hydrogen' has      0 atoms in      0 residues - defined by dictionary file
      set:         'nonHydrogen' has   6138 atoms in   1282 residues - defined by dictionary file
      set:             'OccZero' has      0 atoms in      0 residues - defined by dictionary file
      set:          'OccNonZero' has   6138 atoms in   1282 residues - defined by dictionary file
      set:             'OccFull' has   6076 atoms in   1278 residues - defined by dictionary file
      set:          'OccNonFull' has     62 atoms in      4 residues - defined by dictionary file
      set:         'ncsautoXcld' has    581 atoms in    581 residues - user specified
      set:        'ncsautoABset' has   2785 atoms in    351 residues - user specified
 No command line arguments '-occ_allhet' or '-occ_restyp' so will not setup automated occcupancy refinement
 
 Interpreting CONSTANT and FREE cards: 
 =====CONSTANT     OCC
 Have found and interpreted      1 CONSTANT cards in the TNT input. 
 Before any COMBINE - Number of atoms with fixed XYZ =     0
 Before any COMBINE - Number of atoms with fixed B   =     0
 Before any COMBINE - Number of atoms with fixed OCC =  6138
 Before any COMBINE - Number of atoms with fixed BIJ =   581
                            cf total number of atoms =  6138
 
 There are no COMBINE cards to be found in the TNT card input 
 
 There are no NOTE BUSTER_COMBINE_RES cards to be found in the TNT card input 

 ############### Working out full connectivity list


 ############### Working out equivalent atoms


 Residue with most atoms given below:
        A|501:O01(LIG)      
        A|501:C02(LIG)      
        A|501:N03(LIG)      
        A|501:C04(LIG)      
        A|501:C05(LIG)      
        A|501:C06(LIG)      
        A|501:C07(LIG)      
        A|501:C08(LIG)      
        A|501:C09(LIG)      
        A|501:C10(LIG)      
        A|501:C11(LIG)      
        A|501:N12(LIG)      
        A|501:C13(LIG)      
        A|501:C14(LIG)      
        A|501:C15(LIG)      


 Have no '-equiv_check_plus' argument - so will take atom type into
 account when checking for equivalence of otherwise identical atoms.
 

 ############### Looking at dihedrals


 ############### Looking at chirals

 NOTE BUSTER_CHIRAL_ANGLECUT card found - read value=    -31.00 degs from it
 NOTE BUSTER_CHIRAL_SIGMA card found - read value=      1.70 degs from it
 RANGE allowed for B variables in refinement:   from      3.00000 to    300.00000

 ############### Preparing for PDB output


 ############### Processing extra restraints explicitly defined

 No 'NOTE BUSTER_TARGET' cards or -target?? args found (used to read coordinates files for soft ncs to external)
 No 'NOTE BUSTER_OCCSUM'     cards found - so not using sum of occupancy restraints
 No 'NOTE BUSTER_DISTANCE'   cards found - so not setting up any additional atom-atom distance restraints
 No 'NOTE BUSTER_UTILANGLE'  cards found - so not setting up any additional restraints on any angle
 No 'NOTE BUSTER_UTILTOR'    cards found - so not setting up any additional sinusoidal restraints on torsions
 
 --- Have some similarity definitions 
     Similarity restraints on RMSD use superposition routines from: 
     Coutsias, E.A., Seok, C., Dill, K.A.(2004)
     "Using quaternions to calculate RMSD", J. Comput. Chem., 25:1849-1857 

 Have found    1 'NOTE BUSTER_SIM_DEFINE' cards 
     Maximum number of chains involved in any SIM group (excluding the template)=     1


 ########## Interpreting card: NOTE BUSTER_SIM_DEFINE ncsautoAB ncsautoABset B
     Number of atoms in template set=  2785
     Number of related chains (other than template) in SIM set=    1
     List of chains to be restrained (excluding template)=   B   
     Pairwise RMSD, XYZ in Angstroms, for SIM group: ncsautoAB
                 A         B   
     A         -----     ----- 
     B         3.165     ----- 
     Overall RMSD,XYZ for the SIM group is     3.165 Angstroms
     Pairwise rms deviation of [individual B's minus their set/chain average] in Angs**2
                 A         B         Bavg
     A         -----     -----     73.07
     B         18.57     -----     58.60
     Overall RMSD,B   for the SIM group is    18.575 Angs**2
     The number of common atoms for the above fits:
                 A         B   
     A         -----     ----- 
     B          2772     ----- 
     (compared to the template set that has    2785 atoms in it)
     List of atoms that are absent in one or more chains for this group: 
                                    A    B   
                    C1      401     +    -
                    O1      401     +    -
                    O2      401     +    -
                    C2      401     +    -
                    C3      401     +    -
                    O7      401     +    -
                    C4      401     +    -
                    C5      401     +    -
                    O3      401     +    -
                    O4      401     +    -
                    C6      401     +    -
                    O5      401     +    -
                    O6      401     +    -
     (key  +  atom present for this chain 
           -  atom absent  for this chain)
 transforms for each of the superpositions orthogonal matrix r11 r12 r13 r21 r22 r23 r31 r32 r33 tx ty tz (PDBSET syntax): 
TRANS_AB -0.877775 -0.478737 -0.017975 -0.478872  0.877876  0.003917  0.013904  0.012046 -0.999831    34.183    32.727    15.527
 reverse transforms 
TRANS_BA -0.877775 -0.478872  0.013904 -0.478737  0.877876  0.012046 -0.017975  0.003917 -0.999831    45.461   -12.552    16.011
 
 Setting up LSSR interatomic distance restraints
 Interpreting card: NOTE BUSTER_SIM_RESTRAIN_LSSR ncsautoAB 6.666
        setting up interatomic distance pair restraints with:
        Dmin=         0.00
        Dmax=         5.50
        sigma=        0.20
        Vmax=         3.00
        weight=       6.67
        number of distance pairs defined for this set is      34775
 Have not supplied either '-sim_swap_equiv' or '-sim_swap_equiv_plus' argument 
   so will not try swaping equivalent atoms to improve NCS and/or target agreement.
 
 NCS by LSSR outlier analysis
 -autoncs option automatic NCS prune routine -------- 
 Residues pruned out by gradient condition:
          A|131     A|132     A|135     A|136     A|145     A|147     A|148
          A|149     A|150     A|151     A|157     A|158     A|159     A|160
          A|165     A|166     A|167     A|168     A|169     A|171     A|172
          A|173     A|174     A|175     A|182     A|187     A|188     A|189
          A|197     A|212     A|215     A|216     A|217     A|219     A|220
          A|253     A|259     A|260     A|264     A|330     A|344     A|345
          B|131     B|132     B|135     B|136     B|145     B|147     B|148
          B|149     B|150     B|151     B|157     B|158     B|159     B|160
          B|165     B|166     B|167     B|168     B|169     B|171     B|172
          B|173     B|174     B|175     B|182     B|187     B|188     B|189
          B|197     B|212     B|215     B|216     B|217     B|219     B|220
          B|253     B|259     B|260     B|264     B|330     B|344     B|345
       
 Residues pruned out by function/max condition:
          A|149!    A|168!    A|169!    A|170     A|171!    A|172!    A|173!
          A|187!    A|188!    A|259!    A|264!   A|ENDA     A|501     B|149!
          B|168!    B|169!    B|170     B|171!    B|172!    B|173!    B|187!
          B|188!    B|259!    B|264!   B|ENDA     B|501 
  
 Total number of residues for which LSSR restraints have been pruned out is       90 out of      700 participating
 Number of LSSR restraints pruned out is      5608 this is   16.1% of the total           34775
  
 -autoncs .Gelly cards suitable for adaptation for manual definition/pruning
           (note auto pruning is done in a slightly different way so exact results may differ slightly)
 -autoncs option .Gelly cards also written to file  auto_gelly_cards.txt
NOTE BUSTER_SET ncsautoXcld = Water    # card added - produced by -autoncs
NOTE BUSTER_SET ncsautoXcld = ncsautoXcld + { A|131 A|132 A|135 A|136 A|145 }    # card added - produced by -autoncs
NOTE BUSTER_SET ncsautoXcld = ncsautoXcld + { A|147 A|148 A|149 A|150 A|151 }    # card added - produced by -autoncs
NOTE BUSTER_SET ncsautoXcld = ncsautoXcld + { A|157 A|158 A|159 A|160 A|165 }    # card added - produced by -autoncs
NOTE BUSTER_SET ncsautoXcld = ncsautoXcld + { A|166 A|167 A|168 A|169 A|170 }    # card added - produced by -autoncs
NOTE BUSTER_SET ncsautoXcld = ncsautoXcld + { A|171 A|172 A|173 A|174 A|175 }    # card added - produced by -autoncs
NOTE BUSTER_SET ncsautoXcld = ncsautoXcld + { A|182 A|187 A|188 A|189 A|197 }    # card added - produced by -autoncs
NOTE BUSTER_SET ncsautoXcld = ncsautoXcld + { A|212 A|215 A|216 A|217 A|219 }    # card added - produced by -autoncs
NOTE BUSTER_SET ncsautoXcld = ncsautoXcld + { A|220 A|253 A|259 A|260 A|264 }    # card added - produced by -autoncs
NOTE BUSTER_SET ncsautoXcld = ncsautoXcld + { A|330 A|344 A|345 A|ENDA A|501 }    # card added - produced by -autoncs
NOTE BUSTER_SET ncsautoABset = Chain_A \ ncsautoXcld    # card added - produced by -autoncs
NOTE BUSTER_SIM_DEFINE ncsautoAB ncsautoABset B    # card added - produced by -autoncs
NOTE BUSTER_SIM_RESTRAIN_LSSR ncsautoAB 6.666    # card added - produced by -autoncs

 LSSR function contribution before pruning     45694.4
                             after pruning     10452.2
 Graph of which residues pruned from LSSR: ncs_pruned.mtv
 Graph of LSSR function outliers ncs_function_outliers.mtv
 Graph of LSSR gradient outliers ncs_gradient_outliers.mtv
 LSSR - analysis of distance difference distribution for NCS
         DeltaD less than 0.5 Angs:  proportion of population= 98.62% average DeltaD of subset     0.012 Angs
 Histogram of LSSR deltaD distribution ncs_histogram.mtv
 
 Interpreting card: NOTE BUSTER_TLS_SET tls1 { A|* }
 Interpreting card: NOTE BUSTER_TLS_SET tls2 { B|* }
 Interpreting card: NOTE BUSTER_TLS_SET tls1 { A|* }
 Interpreting card: NOTE BUSTER_TLS_T   tls1     -1.42739E-01 -2.31034E-02  1.80298E-01 -3.76226E-02  2.26092E-02  1.74795E-02
 Interpreting card: NOTE BUSTER_TLS_L   tls1      1.45453E+00  1.61782E+00  8.45083E-01  9.10505E-02 -6.05438E-01  5.77972E-01
 Interpreting card: NOTE BUSTER_TLS_S   tls1      2.23750E-01 -2.44115E-01  3.38595E-01 -6.35630E-02  3.30303E-03 -2.70940E-01 -8.61559E-02 -8.56052E-02
 Interpreting card: NOTE BUSTER_TLS_O   tls1     -2.33111E+01 -8.37601E+01  1.13266E+01
 Interpreting card: NOTE BUSTER_TLS_FIX tls1 ALL
 Interpreting card: NOTE BUSTER_TLS_SET tls2 { B|* }
 Interpreting card: NOTE BUSTER_TLS_T   tls2     -1.96623E-01 -1.35074E-01  3.50236E-01  1.18743E-02  4.75488E-02 -1.48386E-02
 Interpreting card: NOTE BUSTER_TLS_L   tls2      1.48927E+00  9.09257E-01  1.09932E+00  7.78817E-01 -9.29773E-01 -7.15920E-01
 Interpreting card: NOTE BUSTER_TLS_S   tls2     -3.97061E-02  1.25383E-01 -1.13212E-01  1.50247E-01  1.99842E-01  3.65144E-02 -6.77258E-02  2.74050E-02
 Interpreting card: NOTE BUSTER_TLS_O   tls2      2.21121E+01 -7.20987E+01  3.46915E+00
 Interpreting card: NOTE BUSTER_TLS_FIX tls2 ALL


 TLS information. Total number of groups:     2


 Group   Natoms    FixRB   FixTLS   Group Tag    Group Spec
     1     2785      Y        Y     tls1    { A|* }
     2     2772      Y        Y     tls2    { B|* }
 
NOTE BUSTER_TLS_SET tls1 { A|* }
NOTE BUSTER_TLS_T   tls1     -1.42739E-01 -2.31034E-02  1.80298E-01 -3.76226E-02  2.26092E-02  1.74795E-02
NOTE BUSTER_TLS_L   tls1      1.45453E+00  1.61782E+00  8.45083E-01  9.10505E-02 -6.05438E-01  5.77972E-01
NOTE BUSTER_TLS_S   tls1      2.23750E-01 -2.44115E-01  3.38595E-01 -6.35630E-02  3.30303E-03 -2.70940E-01 -8.61559E-02 -8.56052E-02
NOTE BUSTER_TLS_O   tls1     -2.33111E+01 -8.37601E+01  1.13266E+01
NOTE BUSTER_TLS_FIX tls1 ALL
 
NOTE BUSTER_TLS_SET tls2 { B|* }
NOTE BUSTER_TLS_T   tls2     -1.96623E-01 -1.35074E-01  3.50236E-01  1.18743E-02  4.75488E-02 -1.48386E-02
NOTE BUSTER_TLS_L   tls2      1.48927E+00  9.09257E-01  1.09932E+00  7.78817E-01 -9.29773E-01 -7.15920E-01
NOTE BUSTER_TLS_S   tls2     -3.97061E-02  1.25383E-01 -1.13212E-01  1.50247E-01  1.99842E-01  3.65144E-02 -6.77258E-02  2.74050E-02
NOTE BUSTER_TLS_O   tls2      2.21121E+01 -7.20987E+01  3.46915E+00
NOTE BUSTER_TLS_FIX tls2 ALL


 TLS: mmCIF group specification begin


#
loop_
_pdbx_refine_tls_group.id
_pdbx_refine_tls_group.refine_tls_id
_pdbx_refine_tls_group.beg_PDB_ins_code
_pdbx_refine_tls_group.beg_auth_asym_id
_pdbx_refine_tls_group.beg_auth_seq_id
_pdbx_refine_tls_group.end_PDB_ins_code
_pdbx_refine_tls_group.end_auth_asym_id
_pdbx_refine_tls_group.end_auth_seq_id
_pdbx_refine_tls_group.selection
_pdbx_refine_tls_group.selection_details
   1   1 . A   -1 . A  346 . '{ A|* }'
   2   1 . A  401 . A  501 . '{ A|* }'
   3   2 . B   -1 . B  346 . '{ B|* }'
   4   2 . B  501 . B  501 . '{ B|* }'
#


 TLS: mmCIF group specification end


 Interpreting card: NOTE BUSTER_ANISO_SET Empty
 Interpreting card: NOTE BUSTER_ANISO_WEIGHT SCA  16.0
 Interpreting card: NOTE BUSTER_ANISO_WEIGHT SPH   0.6    0.6
 Interpreting card: NOTE BUSTER_ANISO_WEIGHT SIM   7.8    4.8    2.6    0
 Interpreting card: NOTE BUSTER_ANISO_WEIGHT RIG  21.5   21.5    3.7    0


 After TLS and ANISO: Number of atoms with fixed/combined xyz =     0
 After TLS and ANISO: Number of atoms with fixed/combined B   =     0
 After TLS and ANISO: Number of atoms with fixed/combined OCC =  6138
 After TLS and ANISO: Number of atoms with fixed/combined BIJ =  6138
 After TLS and ANISO: Number of atoms in a TLS group          =  5557
                                     cf total number of atoms =  6138
 

 ############### Setup state vector

 No free_vect_ulim_xyz argument found: using default of  100.000
 No free_vect_llim_xyz argument found: using default of -100.000
 Number of parameters added for TLS description                 =       0
 Number of parameters to describe molecular conformation        =   24552

 ############### Setup free vector

 Number of free parameters to be adjusted in refinement   =   24552

 ############### Setting up own geometry


 No command line argument '-tnt_fn' so will use gelly to do geometry function calculations

 ============= Call TNT functions


 ============= Get TNT symmetry information

 
      Getting symmetry operators from TNT. 
          gelly will classify symmetry using pdb-like convention: 
               SYMOP   SYMMETRY
              NNNMMM   OPERATOR
                1555   X,Y,Z
                2555   -Y,X-Y,Z
                3555   -X+Y,-X,Z
                4555   -X,-Y,1/2+Z
                5555   Y,-X+Y,1/2+Z
                6555   X-Y,X,1/2+Z
          where NNN -> operator number and MMM -> translation vector
 

 ============= Own Gelly geometry/restraints setup


 ----------- Identify bond-types


    Setting up GEOMBONDREC_TYPE_HASH* to be able to map a given bond type (e.g.
    "single") to an internal integer (GBOND_TYPE=1) etc.

    Using default  GEOMBONDREC_TYPE_HASH_STR='SING* 1 DOUB* 2 TRIP* 3 AROM* 4 DELO* 4 METAL* 5':
    to override default use a NOTE BUSTER_GEOMBONDREC_TYPE_HASH_STR gelly card.

 ----------- Classify torsion into peptide omega and other


 ----------- Setup of non-bonded terms

 Ideal-distance contact term setup:
    Restraint function constants (normally these values are from NOTE BUSTER_IDEAL_CONTACT_* cards)
    Sigma for general contacts      0.200 Angs
    Sigma for 1-4 contacts          0.100 Angs (contacts across a torsion)
    Sigma for 1-5 contacts          0.150 Angs
    Sigma for 1-N contacts          0.200 Angs
    Sigma for HBOND pairs           0.100 Angs (contacts between HB_types A and D or B's)
    Contact distance increment for each atom in HBOND          -0.150 Angs 
    Contact distance increment for H-bond acceptor              0.100 Angs 
    Contact distance increment for 1-4 with an O atom          -0.050 Angs 
    Contact distance increment for 1-4 with an N atom          -0.180 Angs 
    Contact distance increment for 1-4 with an C atom          -0.370 Angs 
    Contact distance increment for 1-4 with an S atom          -0.150 Angs 
    Contact distance increment for 1-4 with an P atom          -0.150 Angs 
    Contact distance increment for 1-4 with any other atom     -0.150 Angs 
    All atoms matched for CCP4 atom type.
    Number of CCP4 atom types used is         23 (H-bound and not H-bound counted separately)

 Setting up short/ideal-distance contacts
   Using gelly special position handling. (For TNT instead rerun with argument '-special_tnt')

   No command line argument '-special_all' specified. We will auto-generate
   EXCLUDE cards only for atom-atom contacts across symmetry (i.e. on special
   position) when this involves a contact within a residue to itself.

   Have set up non-bonded exclusions - 1-3 contacts
   Atom A|27:CA         makes the most 1-3 contacts:  6

   Have set up non-bonded exclusions - 1-4 contacts
   Atom A|27:N          makes the most 1-4 contacts:  7

   Have set up non-bonded exclusions - 1-5 contacts
   Atom A|28:C.A        makes the most 1-5 contacts: 10

 ############################################################################################################
 ###################################################### setting up contact terms
 ############################################################################################################


 ============= Call Gelly versions of TNT functions

 WARNING: 1-4 contact without 1-(2,3)-4 data for A|401:O2(CIT)        and A|401:C3(CIT)        X-X
 WARNING: 1-4 contact without 1-(2,3)-4 data for A|401:C2(CIT)        and A|401:O6(CIT)        X-X
 WARNING: 1-4 contact without 1-(2,3)-4 data for A|401:O7(CIT)        and A|401:O5(CIT)        X-X
 WARNING: 1-4 contact without 1-(2,3)-4 data for A|401:C4(CIT)        and A|401:O6(CIT)        X-X
 WARNING: 1-4 contact without 1-(2,3)-4 data for A|501:O01(LIG)       and A|501:C15(LIG)       X-X
 WARNING: 1-4 contact without 1-(2,3)-4 data for A|501:C02(LIG)       and A|501:C09(LIG)       X-X
 WARNING: 1-4 contact without 1-(2,3)-4 data for A|501:C04(LIG)       and A|501:C11(LIG)       X-X
 WARNING: 1-4 contact without 1-(2,3)-4 data for A|501:C05(LIG)       and A|501:N12(LIG)       X-X

       TERM      weight      number      TNT_fn    Gelly_fn        diff
       BOND       2.000        5676    1645.689    1645.689       0.000
       ANGL       2.000        7707    6581.435    6581.435       0.000
       IMPR       0.000           0       0.000       0.000       0.000
       TORS       2.000        1979    2963.223    2963.223      -0.000
       TRIG       2.000           0       0.000       0.000       0.000
       PLAN       5.000         966    3688.121    3688.121      -0.000
       CONT       5.000           0    1626.312       0.000    1626.312
       BCOR      10.000        5630    6611.025    5743.219     867.806


 ============= Call Gelly geometry functions

   Switching torsion functional form to sinusoidal rather than split harmonic functional.
      if you want this not to happen then rerun with '-torsharm'
      Weight for sinusoidal torsion=     2.000
   BCOR function/gradient will be split between H and non-H atoms (as for any atom pair). Use '-bcorrel_all_in_hydrogens' to change this.

 ----------- Fix B-correlation restraints

 
 Checking for bonds that do not have a bcorrel restraint defined
 We will wipe all existing BCORrels if OCC=0.0 atom(s) is/are involved. This can be' switched off via the '-bcorrel_nowipe_zeroocc' flag.
 We will wipe all existing BCORrels if only non-hydrogen atom(s) with fixed B(iso) are involved. This can be switched off via the '-bcorrel_nowipe_fixb_nonhydrogens' flag.
 We will keep all existing BCORrels if hydrogen atom(s) with fixed B(iso) is/are involved. This can be switched off via the '-bcorrel_wipe_fixb_hydrogens' flag.
 No -bcorrel_missing_sigma found. So will set sigma to     2.5 Ang**2 for any bond with no existing BCORrel
 No -bcorrel_missing_sigma_hydrogens found. So will set sigma to     1.0 Ang**2 for any bond with no existing BCORrel involving hydrogens

 No active BCORels found involving OCC=0.0 atom(s)


 No active BCORels found involving fixed-B (only) non-hydrogen atom(s)

    add a default BCOR for bond A|401:C1(CIT) A|401:C2(CIT)
    add a default BCOR for bond A|401:C1(CIT) A|401:O1(CIT)
    add a default BCOR for bond A|401:C1(CIT) A|401:O2(CIT)
    add a default BCOR for bond A|401:C2(CIT) A|401:C3(CIT)
    add a default BCOR for bond A|401:C3(CIT) A|401:C4(CIT)
    add a default BCOR for bond A|401:C3(CIT) A|401:C6(CIT)
    add a default BCOR for bond A|401:C3(CIT) A|401:O7(CIT)
    add a default BCOR for bond A|401:C4(CIT) A|401:C5(CIT)
    add a default BCOR for bond A|401:C5(CIT) A|401:O3(CIT)
    add a default BCOR for bond A|401:C5(CIT) A|401:O4(CIT)
    add a default BCOR for bond A|401:C6(CIT) A|401:O5(CIT)
    add a default BCOR for bond A|401:C6(CIT) A|401:O6(CIT)
    add a default BCOR for bond A|501:O01(LIG) A|501:C02(LIG)
    add a default BCOR for bond A|501:C02(LIG) A|501:N03(LIG)
    add a default BCOR for bond A|501:C02(LIG) A|501:C11(LIG)
    add a default BCOR for bond A|501:N03(LIG) A|501:C04(LIG)
    add a default BCOR for bond A|501:C04(LIG) A|501:C05(LIG)
    add a default BCOR for bond A|501:C04(LIG) A|501:C09(LIG)
    add a default BCOR for bond A|501:C05(LIG) A|501:C06(LIG)
    add a default BCOR for bond A|501:C05(LIG) A|501:C10(LIG)
    add a default BCOR for bond A|501:C06(LIG) A|501:C07(LIG)
    add a default BCOR for bond A|501:C07(LIG) A|501:C08(LIG)
    add a default BCOR for bond A|501:C08(LIG) A|501:C09(LIG)
    add a default BCOR for bond A|501:C10(LIG) A|501:C11(LIG)
    add a default BCOR for bond A|501:C11(LIG) A|501:N12(LIG)
    add a default BCOR for bond A|501:C11(LIG) A|501:C15(LIG)
    add a default BCOR for bond A|501:N12(LIG) A|501:C13(LIG)
    add a default BCOR for bond A|501:C13(LIG) A|501:C14(LIG)
    add a default BCOR for bond A|501:C14(LIG) A|501:C15(LIG)
    add a default BCOR for bond B|501:O01(LIG) B|501:C02(LIG)
    add a default BCOR for bond B|501:C02(LIG) B|501:N03(LIG)
    add a default BCOR for bond B|501:C02(LIG) B|501:C11(LIG)
    add a default BCOR for bond B|501:N03(LIG) B|501:C04(LIG)
    add a default BCOR for bond B|501:C04(LIG) B|501:C05(LIG)
    add a default BCOR for bond B|501:C04(LIG) B|501:C09(LIG)
    add a default BCOR for bond B|501:C05(LIG) B|501:C06(LIG)
    add a default BCOR for bond B|501:C05(LIG) B|501:C10(LIG)
    add a default BCOR for bond B|501:C06(LIG) B|501:C07(LIG)
    add a default BCOR for bond B|501:C07(LIG) B|501:C08(LIG)
    add a default BCOR for bond B|501:C08(LIG) B|501:C09(LIG)
    add a default BCOR for bond B|501:C10(LIG) B|501:C11(LIG)
    add a default BCOR for bond B|501:C11(LIG) B|501:N12(LIG)
    add a default BCOR for bond B|501:C11(LIG) B|501:C15(LIG)
    add a default BCOR for bond B|501:N12(LIG) B|501:C13(LIG)
    add a default BCOR for bond B|501:C13(LIG) B|501:C14(LIG)
    add a default BCOR for bond B|501:C14(LIG) B|501:C15(LIG)

 Have added a total of        46 BCORrels for bonds which do not already have a BCORrel


 No bonds (for potential BCORrel addition) were skipped due to both atoms having constant/fixed B(iso)


 ----------- Deactivate hydrogen-free planes if hydrogen atoms present

 A total of       0 planes have been deactivated because they are duplicated hydrogen-free and with-hydrogen forms.

 ----------- Handle peptide omega angle(s)

      Torsion angles split into peptide omega and other
         Number of peptide omega angles is       698
         Number of other torsion is             1281
      Peptide omega angles are monitored rather than directly restrained (PLANE used instead).

 ----------- Fix cis-PRO

 Now apply the fix to get correct restraints for cis PRO residues
   Have got      11 bonds records and      26 angles
   from GEOMETRY cards for the special PROC residue
   Weight for ideal-distance contact term=     4.000

 ----------- Prepare SSBOND/LINK for output PDB file


 ----------- Prepare for QM


 ----------- Prepare symmetry in utility distance restraints


 ############### Setting up Rhofit contacts (if needed)

 No ligand-(fixed protein) contact term will be used

 ############### Working out initial function and gradient


   MaxLik Scaling Cycle 0002
 
 Initial functional value = 5.422696E+05 rms gradient=      14.4025
 X-ray contribution to function value = 5.010324E+05
 Geometry contribution (inc ncs)      =      41237.2
 Geometry function summary
     term                           number     weight       rms        GooF      function     fn/numb
     ================================================================================================
     BOND bond lengths (angs)       5676       2.000      0.0076       0.381      1645.69       0.290
     ANGL bond angles (degs)        7707       2.000        1.10       0.653      6581.43       0.854
     TORS split-harmonic tors       1281       0.000       17.15       1.075         0.00       0.000
     SINTOR sinusoidal tors         1281       2.000       17.15                  2232.38       1.743
     PLAN general plane (angs)       966       5.000      0.0175       0.874      3688.12       3.818
     BCORrelations (angs^2)         5676      10.000       0.891       0.318      5744.04       1.012
     CONT (bad contacts)               0       5.000                                 0.00
     IDEAL(ideal-dist contact)      5073       4.000                             10835.18
     CHIRAL (gelly semiharmon)       740       5.000                                58.08
     SIM similarity restraint for NCS and/or TARGET                              10452.24
     OCCSUM restraint on sum of occupanies                                           0.00       as none defined
     DISTAN utility restraints interatomic distance                                  0.00       as none defined
     UTILANGLE utility angle restraints                                              0.00       as none defined
     UTILTOR utility restraints on torsion angles                                    0.00       as none defined
     Peptide omega torsion angles                           3.27 degs - monitor only
 
 WARNING: Have |delta/sigma| deviations > 5.0 sigma. Number of outliers for each term: 
 WARNING:         3 bond angles.  Worst is    18.9 sigs    63.32 degs A|501:C10=C05=C04 (LIG)
 WARNING:         1 planes.       Worst is    12.4 sigs     0.25 Angs A|501:N03=C04=C05=C06=C07=C08=C09=C10 (LIG)
 WARNING:         1 idealD contacts. Worst     7.3 sigs     2.21 Angs A|501:C09=C11 (LIG)

 Weighted rms      0.0076         1.098  (temporary rms bond and angle for BUSTER to pick up!)
 Weighted intra-residual rms(bond) for StdProtein (excluding H)      0.0072
 Weighted intra-residual rms(bond) for Buffer     (excluding H)      0.0038

 ############### Running initial check


 Initial Geometry Sanity Check

 Check for duplicate bonds: no duplicates found
 
 Check for duplicate or missing bond angles: all are OK
 Check for bond between an atom and itself not involving symmetry:  all are OK
 *** WARNING: There are 1 bad contacts where Delta/sigma is greater than      6.5
 *** WARNING: Looking at each one in turn to see whether it is likely to indicate a setup problem with geometry restraints.

        1 dist=   2.21 idealD=   3.67 sigma=   0.20 abs(Delta/sigma)=    7.28
          Between atoms   A|501:C09 (LIG)         and   A|501:C11 (LIG)         (C-C)
          occupancies=     0.44        0.44
 [check-033]
          This is a contact within a residue - so probably just a distorted
          residue?
 GEOMETRY CHECK OVERALL:  WARN


 ############### Creating geometry/screen report


 ############### Option "tao" - run Tao optimizer

 TAO: Toolkit for Advanced Optimization. Reference: 
     Munson, T., Sarich, J., Wild, S., Benson, S., McInnes, L.C. (2018)
     "TAO User Manual (Revision 3.9)", Mathematics and Computer Science
     Division, Argonne National Laboratory
     ANL/MCS-TM-322, http://www.mcs.anl.gov/petsc/
 
 Have found -keeppdb flag so will write out a .pdb file every 1000000 its during opt
 Will apply TAO optimization 
 Maximum number of iterations      100
 Convergence test on Grms       4.0000 (-glim)
 Limit on rms displacement to original position (rmsD2init): NONE test inactive (-dlim)

    analysing argument no 0 : -autoncs with j=0
    analysing argument no 1 : -hydrogens_input_occ2zero with j=0
    analysing argument no 2 : -hydrogens_output_occ2heavy with j=0
    analysing argument no 3 : -screen with j=0
    analysing argument no 4 : 100 with j=0
    analysing argument no 5 : -screen_sigma with j=0
    analysing argument no 6 : 3.0 with j=0
    analysing argument no 7 : -keeppdb with j=0
    analysing argument no 8 : 1000000 with j=0
    analysing argument no 9 : -taomonitor_verbose with j=0
    found argument starting with "-tao" : -taomonitor_verbose
    analysing argument no 10 : 2 with j=1
    found value after -tao/-options_/-viewer_ argument : "2"
 PETSC/TAO: setting option "-taomonitor_verbose" = "2"


   MaxLik Scaling Cycle 0003
   Iters     Tot_Funct    Xray_Funct    Geom_Funct          Grms     rmsD2init     maxD2init             R         Rfree           LLG       LLGfree     rms(bond)    rms(angle)       Opttag_H
   Iters             K   B_IMPF_FRAG        K_MISS   B_TEMP_MISS   K_IMPF_MISS   B_IMPF_MISS        K_SOLV   B_TEMP_SOLV   K_IMPF_SOLV   B_IMPF_SOLV        B_TEMP     B_TEMP_11     B_TEMP_12     B_TEMP_22     B_TEMP_13     B_TEMP_23     B_TEMP_33      K_WILSON      B_WILSON      FGCYCLES       Scatag_H
   Iters          BOND         ANGLE      IMPROPER       TORSION    SINTORSION      TRIGONAL        PLANAR  BCORRELATION       CONTACT     IDEALDIST        CHIRAL    SIMILARITY       OCCSSUM  UTILDISTANCE     UTILANGLE   UTILTORSION        RHOFIT        ADP-01        ADP-02        ADP-03        ADP-04        ADP-05        ADP-06        ADP-07        ADP-08        ADP-09        ADP-10      RHOFITPC            QM       Funtag_H
       0  5.422696E+05  5.010324E+05       41237.2       14.4025  0.000000E+00  0.000000E+00      0.212069      0.230327  0.000000E+00  0.000000E+00    0.00764007       1.09813       Opttag_D
       0       46.4741       2.07497       1.00000  0.000000E+00       1.00000  0.000000E+00      0.794616       63.7987       1.00000       25.6156  0.000000E+00       1.03413  0.000000E+00       1.03413  0.000000E+00  0.000000E+00      -2.06825       46.8739       1.38831        2       Scatag_D
       0       1645.69       6581.43  0.000000E+00  0.000000E+00       2232.38  0.000000E+00       3688.12       5744.04  0.000000E+00       10835.2       58.0842       10452.2 -1.000000E+10 -1.000000E+10 -1.000000E+10 -1.000000E+10 -1.000000E+10  0.000000E+00  0.000000E+00  0.000000E+00  0.000000E+00  0.000000E+00  0.000000E+00  0.000000E+00  0.000000E+00  0.000000E+00  0.000000E+00  0.000000E+00  0.000000E+00       Funtag_D
   Geometry Restraint Deviations SCREEN Information

 

 WARNING: have displacement of 32.90 Angs from initial (> 15.00 Angs)
          for variable number= 8174 (type Y        ; returned function value as   3.551714E+011 )
          Involving atom A|258:CG

 An atom has moved more than     1.50 A from the initial/last
 position - if this was an accepted trial point, probably need to update the
 non-bonded contact list.


   MaxLik Scaling Cycle 0004

   MaxLik Scaling Cycle 0005

   MaxLik Scaling Cycle 0006

   Wilson plot

   Statistics in resolution bins after refinement cycle 0001
```


|  |  |
| --- | --- |
|  | - Rxpct factors plot- Rxpct factors table- <Log-Lik Gain>- Ln Sigma A- Correlation Coefficients |


```
   Iters     Tot_Funct    Xray_Funct    Geom_Funct          Grms     rmsD2init     maxD2init             R         Rfree           LLG       LLGfree     rms(bond)    rms(angle)
   Iters             K   B_IMPF_FRAG        K_MISS   B_TEMP_MISS   K_IMPF_MISS   B_IMPF_MISS        K_SOLV   B_TEMP_SOLV   K_IMPF_SOLV   B_IMPF_SOLV        B_TEMP     B_TEMP_11     B_TEMP_12     B_TEMP_22     B_TEMP_13     B_TEMP_23     B_TEMP_33      K_WILSON      B_WILSON      FGCYCLES
   Iters          BOND         ANGLE      IMPROPER       TORSION    SINTORSION      TRIGONAL        PLANAR  BCORRELATION       CONTACT     IDEALDIST        CHIRAL    SIMILARITY       OCCSSUM  UTILDISTANCE     UTILANGLE   UTILTORSION        RHOFIT        ADP-01        ADP-02        ADP-03        ADP-04        ADP-05        ADP-06        ADP-07        ADP-08        ADP-09        ADP-10      RHOFITPC            QM
       1  5.422273E+05  5.008705E+05       41356.9       15.2863  3.684992E-04    0.00329963      0.211979      0.230282  3.589905E-04  5.485229E-05    0.00769212       1.09862       Opttag_D
       1       46.4753       2.07072       1.00000  0.000000E+00       1.00000  0.000000E+00      0.794605       63.7853       1.00000       25.6180  0.000000E+00       1.03080  0.000000E+00       1.03080  0.000000E+00  0.000000E+00      -2.06160       46.8739       1.38831        3       Scatag_D
       1       1668.19       6587.41  0.000000E+00  0.000000E+00       2232.33  0.000000E+00       3693.80       5831.84  0.000000E+00       10834.6       57.7694       10451.0 -1.000000E+10 -1.000000E+10 -1.000000E+10 -1.000000E+10 -1.000000E+10  0.000000E+00  0.000000E+00  0.000000E+00  0.000000E+00  0.000000E+00  0.000000E+00  0.000000E+00  0.000000E+00  0.000000E+00  0.000000E+00  0.000000E+00  0.000000E+00       Funtag_D
   Geometry Restraint Deviations SCREEN Information

 

   MaxLik Scaling Cycle 0007

   Wilson plot

   Statistics in resolution bins after refinement cycle 0002
```


|  |  |
| --- | --- |
|  | - Rxpct factors plot- Rxpct factors table- <Log-Lik Gain>- Ln Sigma A- Correlation Coefficients |


```
   Iters     Tot_Funct    Xray_Funct    Geom_Funct          Grms     rmsD2init     maxD2init             R         Rfree           LLG       LLGfree     rms(bond)    rms(angle)
   Iters             K   B_IMPF_FRAG        K_MISS   B_TEMP_MISS   K_IMPF_MISS   B_IMPF_MISS        K_SOLV   B_TEMP_SOLV   K_IMPF_SOLV   B_IMPF_SOLV        B_TEMP     B_TEMP_11     B_TEMP_12     B_TEMP_22     B_TEMP_13     B_TEMP_23     B_TEMP_33      K_WILSON      B_WILSON      FGCYCLES
   Iters          BOND         ANGLE      IMPROPER       TORSION    SINTORSION      TRIGONAL        PLANAR  BCORRELATION       CONTACT     IDEALDIST        CHIRAL    SIMILARITY       OCCSSUM  UTILDISTANCE     UTILANGLE   UTILTORSION        RHOFIT        ADP-01        ADP-02        ADP-03        ADP-04        ADP-05        ADP-06        ADP-07        ADP-08        ADP-09        ADP-10      RHOFITPC            QM
       2  5.421518E+05  5.005535E+05       41598.4       17.3199    0.00130862     0.0108175      0.211753      0.230155    0.00106106  1.578501E-04    0.00783714       1.10077       Opttag_D
       2       46.4834       2.06403       1.00000  0.000000E+00       1.00000  0.000000E+00      0.794600       63.8047       1.00000       25.5817  0.000000E+00       1.02348  0.000000E+00       1.02348  0.000000E+00  0.000000E+00      -2.04696       46.8739       1.38831        1       Scatag_D
       2       1731.68       6613.20  0.000000E+00  0.000000E+00       2232.03  0.000000E+00       3714.82       5955.24  0.000000E+00       10835.1       58.0203       10458.2 -1.000000E+10 -1.000000E+10 -1.000000E+10 -1.000000E+10 -1.000000E+10  0.000000E+00  0.000000E+00  0.000000E+00  0.000000E+00  0.000000E+00  0.000000E+00  0.000000E+00  0.000000E+00  0.000000E+00  0.000000E+00  0.000000E+00  0.000000E+00       Funtag_D
   Geometry Restraint Deviations SCREEN Information

 
       3  5.420956E+05  5.002837E+05       41811.9       15.7926    0.00228112     0.0211841      0.211552      0.230042    0.00165851  2.616862E-04    0.00795979       1.10297       Opttag_D
       3       46.4911       2.05873       1.00000  0.000000E+00       1.00000  0.000000E+00      0.794610       63.8282       1.00000       25.5386  0.000000E+00       1.01860  0.000000E+00       1.01860  0.000000E+00  0.000000E+00      -2.03720       46.8739       1.38831        1       Scatag_D
       3       1786.31       6639.64  0.000000E+00  0.000000E+00       2231.85  0.000000E+00       3737.63       6044.47  0.000000E+00       10840.1       58.4912       10473.5 -1.000000E+10 -1.000000E+10 -1.000000E+10 -1.000000E+10 -1.000000E+10  0.000000E+00  0.000000E+00  0.000000E+00  0.000000E+00  0.000000E+00  0.000000E+00  0.000000E+00  0.000000E+00  0.000000E+00  0.000000E+00  0.000000E+00  0.000000E+00       Funtag_D
       4  5.420521E+05  5.001203E+05       41931.8       12.8316    0.00295363     0.0295476      0.211426      0.229970    0.00202032  3.566597E-04    0.00801082       1.10430       Opttag_D
       4       46.4964       2.05577       1.00000  0.000000E+00       1.00000  0.000000E+00      0.794620       63.8470       1.00000       25.5069  0.000000E+00       1.01583  0.000000E+00       1.01583  0.000000E+00  0.000000E+00      -2.03165       46.8739       1.38831        1       Scatag_D
       4       1809.28       6655.62  0.000000E+00  0.000000E+00       2231.85  0.000000E+00       3753.42       6092.94  0.000000E+00       10846.4       58.8445       10483.4 -1.000000E+10 -1.000000E+10 -1.000000E+10 -1.000000E+10 -1.000000E+10  0.000000E+00  0.000000E+00  0.000000E+00  0.000000E+00  0.000000E+00  0.000000E+00  0.000000E+00  0.000000E+00  0.000000E+00  0.000000E+00  0.000000E+00  0.000000E+00       Funtag_D
       5  5.419953E+05  4.998702E+05       42125.2       13.0133    0.00418148     0.0459887      0.211226      0.229852    0.00257436  5.048088E-04    0.00806819       1.10668       Opttag_D
       5       46.5062       2.05166       1.00000  0.000000E+00       1.00000  0.000000E+00      0.794644       63.8836       1.00000       25.4497  0.000000E+00       1.01175  0.000000E+00       1.01175  0.000000E+00  0.000000E+00      -2.02350       46.8739       1.38831        1       Scatag_D
       5       1835.29       6684.35  0.000000E+00  0.000000E+00       2231.99  0.000000E+00       3780.63       6174.37  0.000000E+00       10861.1       59.5086       10497.9 -1.000000E+10 -1.000000E+10 -1.000000E+10 -1.000000E+10 -1.000000E+10  0.000000E+00  0.000000E+00  0.000000E+00  0.000000E+00  0.000000E+00  0.000000E+00  0.000000E+00  0.000000E+00  0.000000E+00  0.000000E+00  0.000000E+00  0.000000E+00       Funtag_D
       6  5.419453E+05  4.995760E+05       42369.4       20.0286    0.00600838     0.0723633      0.210976      0.229671    0.00322588  7.423025E-04    0.00812085       1.11029       Opttag_D
       6       46.5217       2.04771       1.00000  0.000000E+00       1.00000  0.000000E+00      0.794671       63.9453       1.00000       25.3694  0.000000E+00       1.00664  0.000000E+00       1.00664  0.000000E+00  0.000000E+00      -2.01328       46.8739       1.38831        1       Scatag_D
       6       1859.33       6728.03  0.000000E+00  0.000000E+00       2232.21  0.000000E+00       3816.96       6273.95  0.000000E+00       10886.4       60.3933       10512.1 -1.000000E+10 -1.000000E+10 -1.000000E+10 -1.000000E+10 -1.000000E+10  0.000000E+00  0.000000E+00  0.000000E+00  0.000000E+00  0.000000E+00  0.000000E+00  0.000000E+00  0.000000E+00  0.000000E+00  0.000000E+00  0.000000E+00  0.000000E+00       Funtag_D
       7  5.418897E+05  4.994697E+05       42420.0       13.8584    0.00697251     0.0881007      0.210876      0.229585    0.00346121  8.515280E-04    0.00811291       1.11102       Opttag_D
       7       46.5298       2.04690       1.00000  0.000000E+00       1.00000  0.000000E+00      0.794682       63.9784       1.00000       25.3298  0.000000E+00       1.00455  0.000000E+00       1.00455  0.000000E+00  0.000000E+00      -2.00909       46.8739       1.38831        1       Scatag_D
       7       1855.69       6736.88  0.000000E+00  0.000000E+00       2232.50  0.000000E+00       3831.79       6289.97  0.000000E+00       10901.9       60.7759       10510.4 -1.000000E+10 -1.000000E+10 -1.000000E+10 -1.000000E+10 -1.000000E+10  0.000000E+00  0.000000E+00  0.000000E+00  0.000000E+00  0.000000E+00  0.000000E+00  0.000000E+00  0.000000E+00  0.000000E+00  0.000000E+00  0.000000E+00  0.000000E+00       Funtag_D
       8  5.418332E+05  4.993845E+05       42448.7       12.6705    0.00819026      0.109905      0.210784      0.229480    0.00365002  9.893774E-04    0.00808395       1.11204       Opttag_D
       8       46.5396       2.04702       1.00000  0.000000E+00       1.00000  0.000000E+00      0.794686       64.0200       1.00000       25.2849  0.000000E+00       1.00229  0.000000E+00       1.00229  0.000000E+00  0.000000E+00      -2.00458       46.8739       1.38831        1       Scatag_D
       8       1842.47       6749.28  0.000000E+00  0.000000E+00       2232.94  0.000000E+00       3844.51       6295.77  0.000000E+00       10922.3       61.1739       10500.3 -1.000000E+10 -1.000000E+10 -1.000000E+10 -1.000000E+10 -1.000000E+10  0.000000E+00  0.000000E+00  0.000000E+00  0.000000E+00  0.000000E+00  0.000000E+00  0.000000E+00  0.000000E+00  0.000000E+00  0.000000E+00  0.000000E+00  0.000000E+00       Funtag_D
       9  5.417840E+05  4.992763E+05       42507.7       15.3926    0.00978102      0.139054      0.210674      0.229361    0.00388958    0.00117142    0.00807096       1.11338       Opttag_D
       9       46.5508       2.04685       1.00000  0.000000E+00       1.00000  0.000000E+00      0.794679       64.0660       1.00000       25.2331  0.000000E+00      0.999374  0.000000E+00      0.999374  0.000000E+00  0.000000E+00      -1.99875       46.8739       1.38831        1       Scatag_D
       9       1836.55       6765.53  0.000000E+00  0.000000E+00       2233.46  0.000000E+00       3857.99       6315.08  0.000000E+00       10946.8       61.7136       10490.6 -1.000000E+10 -1.000000E+10 -1.000000E+10 -1.000000E+10 -1.000000E+10  0.000000E+00  0.000000E+00  0.000000E+00  0.000000E+00  0.000000E+00  0.000000E+00  0.000000E+00  0.000000E+00  0.000000E+00  0.000000E+00  0.000000E+00  0.000000E+00       Funtag_D
      10  5.417284E+05  4.991398E+05       42588.6       14.0310     0.0119549      0.179574      0.210534      0.229234    0.00419198    0.00135466    0.00807437       1.11533       Opttag_D
      10       46.5642       2.04647       1.00000  0.000000E+00       1.00000  0.000000E+00      0.794658       64.1207       1.00000       25.1695  0.000000E+00      0.995435  0.000000E+00      0.995435  0.000000E+00  0.000000E+00      -1.99087       46.8739       1.38831        1       Scatag_D
      10       1838.10       6789.24  0.000000E+00  0.000000E+00       2234.23  0.000000E+00       3873.37       6330.13  0.000000E+00       10978.8       62.4351       10482.3 -1.000000E+10 -1.000000E+10 -1.000000E+10 -1.000000E+10 -1.000000E+10  0.000000E+00  0.000000E+00  0.000000E+00  0.000000E+00  0.000000E+00  0.000000E+00  0.000000E+00  0.000000E+00  0.000000E+00  0.000000E+00  0.000000E+00  0.000000E+00       Funtag_D
      11  5.416820E+05  4.990661E+05       42615.9       12.5111     0.0132176      0.205578      0.210466      0.229182    0.00435511    0.00142269    0.00808121       1.11601       Opttag_D
      11       46.5697       2.04581       1.00000  0.000000E+00       1.00000  0.000000E+00      0.794622       64.1432       1.00000       25.1404  0.000000E+00      0.993475  0.000000E+00      0.993475  0.000000E+00  0.000000E+00      -1.98695       46.8739       1.38831        1       Scatag_D
      11       1841.22       6797.59  0.000000E+00  0.000000E+00       2234.66  0.000000E+00       3876.97       6333.58  0.000000E+00       10993.6       62.7503       10475.6 -1.000000E+10 -1.000000E+10 -1.000000E+10 -1.000000E+10 -1.000000E+10  0.000000E+00  0.000000E+00  0.000000E+00  0.000000E+00  0.000000E+00  0.000000E+00  0.000000E+00  0.000000E+00  0.000000E+00  0.000000E+00  0.000000E+00  0.000000E+00       Funtag_D
      12  5.416359E+05  4.989931E+05       42642.8       11.5525     0.0144266      0.232008      0.210405      0.229154    0.00451684    0.00145275    0.00809651       1.11672       Opttag_D
      12       46.5735       2.04478       1.00000  0.000000E+00       1.00000  0.000000E+00      0.794575       64.1588       1.00000       25.1174  0.000000E+00      0.991941  0.000000E+00      0.991941  0.000000E+00  0.000000E+00      -1.98388       46.8739       1.38831        1       Scatag_D
      12       1848.20       6806.19  0.000000E+00  0.000000E+00       2235.09  0.000000E+00       3877.69       6337.90  0.000000E+00       11004.5       62.9529       10470.3 -1.000000E+10 -1.000000E+10 -1.000000E+10 -1.000000E+10 -1.000000E+10  0.000000E+00  0.000000E+00  0.000000E+00  0.000000E+00  0.000000E+00  0.000000E+00  0.000000E+00  0.000000E+00  0.000000E+00  0.000000E+00  0.000000E+00  0.000000E+00       Funtag_D
      13  5.415973E+05  4.989197E+05       42677.5       13.2467     0.0158372      0.263348      0.210341      0.229132    0.00467926    0.00144927    0.00811109       1.11761       Opttag_D
      13       46.5780       2.04383       1.00000  0.000000E+00       1.00000  0.000000E+00      0.794517       64.1773       1.00000       25.0928  0.000000E+00      0.990770  0.000000E+00      0.990770  0.000000E+00  0.000000E+00      -1.98154       46.8739       1.38831        1       Scatag_D
      13       1854.86       6817.07  0.000000E+00  0.000000E+00       2235.68  0.000000E+00       3877.72       6345.77  0.000000E+00       11016.4       63.0774       10466.9 -1.000000E+10 -1.000000E+10 -1.000000E+10 -1.000000E+10 -1.000000E+10  0.000000E+00  0.000000E+00  0.000000E+00  0.000000E+00  0.000000E+00  0.000000E+00  0.000000E+00  0.000000E+00  0.000000E+00  0.000000E+00  0.000000E+00  0.000000E+00       Funtag_D
      14  5.415554E+05  4.988670E+05       42688.4       10.6900     0.0171045      0.292411      0.210295      0.229113    0.00479597    0.00143454    0.00811720       1.11823       Opttag_D
      14       46.5822       2.04330       1.00000  0.000000E+00       1.00000  0.000000E+00      0.794460       64.1961       1.00000       25.0735  0.000000E+00      0.990404  0.000000E+00      0.990404  0.000000E+00  0.000000E+00      -1.98081       46.8739       1.38831        1       Scatag_D
      14       1857.65       6824.65  0.000000E+00  0.000000E+00       2236.35  0.000000E+00       3876.02       6340.43  0.000000E+00       11025.8       63.0830       10464.4 -1.000000E+10 -1.000000E+10 -1.000000E+10 -1.000000E+10 -1.000000E+10  0.000000E+00  0.000000E+00  0.000000E+00  0.000000E+00  0.000000E+00  0.000000E+00  0.000000E+00  0.000000E+00  0.000000E+00  0.000000E+00  0.000000E+00  0.000000E+00       Funtag_D
      15  5.415159E+05  4.988182E+05       42697.7       10.8249     0.0185352      0.324168      0.210246      0.229077    0.00490412    0.00144868    0.00810883       1.11901       Opttag_D
      15       46.5888       2.04337       1.00000  0.000000E+00       1.00000  0.000000E+00      0.794411       64.2241       1.00000       25.0493  0.000000E+00      0.990081  0.000000E+00      0.990081  0.000000E+00  0.000000E+00      -1.98016       46.8739       1.38831        1       Scatag_D
      15       1853.83       6834.10  0.000000E+00  0.000000E+00       2237.26  0.000000E+00       3875.44       6334.80  0.000000E+00       11038.7       63.1948       10460.4 -1.000000E+10 -1.000000E+10 -1.000000E+10 -1.000000E+10 -1.000000E+10  0.000000E+00  0.000000E+00  0.000000E+00  0.000000E+00  0.000000E+00  0.000000E+00  0.000000E+00  0.000000E+00  0.000000E+00  0.000000E+00  0.000000E+00  0.000000E+00       Funtag_D
      16  5.414779E+05  4.987593E+05       42718.6       13.1335     0.0206957      0.371364      0.210180      0.229019    0.00503453    0.00147263    0.00810328       1.12018       Opttag_D
      16       46.5990       2.04390       1.00000  0.000000E+00       1.00000  0.000000E+00      0.794348       64.2666       1.00000       25.0129  0.000000E+00      0.989788  0.000000E+00      0.989788  0.000000E+00  0.000000E+00      -1.97958       46.8739       1.38831        1       Scatag_D
      16       1851.29       6848.47  0.000000E+00  0.000000E+00       2238.76  0.000000E+00       3874.89       6324.49  0.000000E+00       11059.5       63.5555       10457.6 -1.000000E+10 -1.000000E+10 -1.000000E+10 -1.000000E+10 -1.000000E+10  0.000000E+00  0.000000E+00  0.000000E+00  0.000000E+00  0.000000E+00  0.000000E+00  0.000000E+00  0.000000E+00  0.000000E+00  0.000000E+00  0.000000E+00  0.000000E+00       Funtag_D
      17  5.414478E+05  4.987271E+05       42720.7       10.6654     0.0218811      0.396905      0.210143      0.228988    0.00510592    0.00148029    0.00809890       1.12066       Opttag_D
      17       46.6047       2.04417       1.00000  0.000000E+00       1.00000  0.000000E+00      0.794313       64.2901       1.00000       24.9940  0.000000E+00      0.989412  0.000000E+00      0.989412  0.000000E+00  0.000000E+00      -1.97882       46.8739       1.38831        1       Scatag_D
      17       1849.29       6854.28  0.000000E+00  0.000000E+00       2239.78  0.000000E+00       3874.89       6311.28  0.000000E+00       11071.1       63.7916       10456.3 -1.000000E+10 -1.000000E+10 -1.000000E+10 -1.000000E+10 -1.000000E+10  0.000000E+00  0.000000E+00  0.000000E+00  0.000000E+00  0.000000E+00  0.000000E+00  0.000000E+00  0.000000E+00  0.000000E+00  0.000000E+00  0.000000E+00  0.000000E+00       Funtag_D
      18  5.414186E+05  4.987016E+05       42717.0       9.29999     0.0227611      0.415709      0.210114      0.228966    0.00516231    0.00148029    0.00809465       1.12084       Opttag_D
      18       46.6092       2.04435       1.00000  0.000000E+00       1.00000  0.000000E+00      0.794289       64.3070       1.00000       24.9801  0.000000E+00      0.988855  0.000000E+00      0.988855  0.000000E+00  0.000000E+00      -1.97771       46.8739       1.38831        1       Scatag_D
      18       1847.35       6856.55  0.000000E+00  0.000000E+00       2240.70  0.000000E+00       3875.19       6300.53  0.000000E+00       11079.1       63.9952       10453.5 -1.000000E+10 -1.000000E+10 -1.000000E+10 -1.000000E+10 -1.000000E+10  0.000000E+00  0.000000E+00  0.000000E+00  0.000000E+00  0.000000E+00  0.000000E+00  0.000000E+00  0.000000E+00  0.000000E+00  0.000000E+00  0.000000E+00  0.000000E+00       Funtag_D
      19  5.413805E+05  4.986385E+05       42741.9       12.1570     0.0247782      0.458906      0.210055      0.228934    0.00530208    0.00140580    0.00810322       1.12154       Opttag_D
      19       46.6186       2.04434       1.00000  0.000000E+00       1.00000  0.000000E+00      0.794227       64.3399       1.00000       24.9527  0.000000E+00      0.987179  0.000000E+00      0.987179  0.000000E+00  0.000000E+00      -1.97436       46.8739       1.38831        1       Scatag_D
      19       1851.26       6865.12  0.000000E+00  0.000000E+00       2242.89  0.000000E+00       3878.06       6293.68  0.000000E+00       11094.5       64.2360       10452.2 -1.000000E+10 -1.000000E+10 -1.000000E+10 -1.000000E+10 -1.000000E+10  0.000000E+00  0.000000E+00  0.000000E+00  0.000000E+00  0.000000E+00  0.000000E+00  0.000000E+00  0.000000E+00  0.000000E+00  0.000000E+00  0.000000E+00  0.000000E+00       Funtag_D
      20  5.413561E+05  4.985583E+05       42797.7       14.8326     0.0274422      0.515733      0.209976      0.228899    0.00547962    0.00131717    0.00812630       1.12279       Opttag_D
      20       46.6314       2.04438       1.00000  0.000000E+00       1.00000  0.000000E+00      0.794147       64.3813       1.00000       24.9207  0.000000E+00      0.984818  0.000000E+00      0.984818  0.000000E+00  0.000000E+00      -1.96964       46.8739       1.38831        1       Scatag_D
      20       1861.82       6880.37  0.000000E+00  0.000000E+00       2245.95  0.000000E+00       3884.24       6290.82  0.000000E+00       11114.4       64.4399       10455.7 -1.000000E+10 -1.000000E+10 -1.000000E+10 -1.000000E+10 -1.000000E+10  0.000000E+00  0.000000E+00  0.000000E+00  0.000000E+00  0.000000E+00  0.000000E+00  0.000000E+00  0.000000E+00  0.000000E+00  0.000000E+00  0.000000E+00  0.000000E+00       Funtag_D
      21  5.413277E+05  4.985494E+05       42778.3       8.78794     0.0275405      0.518077      0.209973      0.228904    0.00549935    0.00129442    0.00812256       1.12259       Opttag_D
      21       46.6321       2.04419       1.00000  0.000000E+00       1.00000  0.000000E+00      0.794140       64.3807       1.00000       24.9218  0.000000E+00      0.984364  0.000000E+00      0.984364  0.000000E+00  0.000000E+00      -1.96873       46.8739       1.38831        1       Scatag_D
      21       1860.11       6877.94  0.000000E+00  0.000000E+00       2246.17  0.000000E+00       3884.95       6280.16  0.000000E+00       11112.0       64.2552       10452.7 -1.000000E+10 -1.000000E+10 -1.000000E+10 -1.000000E+10 -1.000000E+10  0.000000E+00  0.000000E+00  0.000000E+00  0.000000E+00  0.000000E+00  0.000000E+00  0.000000E+00  0.000000E+00  0.000000E+00  0.000000E+00  0.000000E+00  0.000000E+00       Funtag_D
      22  5.413031E+05  4.985233E+05       42779.8       7.86309     0.0282617      0.533810      0.209955      0.228897    0.00555725    0.00126532    0.00812353       1.12277       Opttag_D
      22       46.6362       2.04405       1.00000  0.000000E+00       1.00000  0.000000E+00      0.794114       64.3890       1.00000       24.9174  0.000000E+00      0.983242  0.000000E+00      0.983242  0.000000E+00  0.000000E+00      -1.96648       46.8739       1.38831        1       Scatag_D
      22       1860.56       6880.20  0.000000E+00  0.000000E+00       2247.16  0.000000E+00       3886.91       6278.32  0.000000E+00       11112.7       64.0485       10449.9 -1.000000E+10 -1.000000E+10 -1.000000E+10 -1.000000E+10 -1.000000E+10  0.000000E+00  0.000000E+00  0.000000E+00  0.000000E+00  0.000000E+00  0.000000E+00  0.000000E+00  0.000000E+00  0.000000E+00  0.000000E+00  0.000000E+00  0.000000E+00       Funtag_D
      23  5.412795E+05  4.984849E+05       42794.6       9.01942     0.0297273      0.565386      0.209922      0.228871    0.00564224    0.00124304    0.00812672       1.12337       Opttag_D
      23       46.6445       2.04424       1.00000  0.000000E+00       1.00000  0.000000E+00      0.794074       64.4082       1.00000       24.9069  0.000000E+00      0.981621  0.000000E+00      0.981621  0.000000E+00  0.000000E+00      -1.96324       46.8739       1.38831        1       Scatag_D
      23       1862.02       6887.56  0.000000E+00  0.000000E+00       2249.10  0.000000E+00       3888.78       6275.70  0.000000E+00       11119.2       64.0213       10448.2 -1.000000E+10 -1.000000E+10 -1.000000E+10 -1.000000E+10 -1.000000E+10  0.000000E+00  0.000000E+00  0.000000E+00  0.000000E+00  0.000000E+00  0.000000E+00  0.000000E+00  0.000000E+00  0.000000E+00  0.000000E+00  0.000000E+00  0.000000E+00       Funtag_D
      24  5.412577E+05  4.984333E+05       42824.4       12.1653     0.0323185      0.620755      0.209875      0.228823    0.00575664    0.00124927    0.00813007       1.12468       Opttag_D
      24       46.6594       2.04500       1.00000  0.000000E+00       1.00000  0.000000E+00      0.794015       64.4409       1.00000       24.8914  0.000000E+00      0.978974  0.000000E+00      0.978974  0.000000E+00  0.000000E+00      -1.95795       46.8739       1.38831        1       Scatag_D
      24       1863.55       6903.62  0.000000E+00  0.000000E+00       2252.58  0.000000E+00       3889.53       6270.75  0.000000E+00       11132.0       64.2006       10448.1 -1.000000E+10 -1.000000E+10 -1.000000E+10 -1.000000E+10 -1.000000E+10  0.000000E+00  0.000000E+00  0.000000E+00  0.000000E+00  0.000000E+00  0.000000E+00  0.000000E+00  0.000000E+00  0.000000E+00  0.000000E+00  0.000000E+00  0.000000E+00       Funtag_D
      25  5.412346E+05  4.984299E+05       42804.6       7.19726     0.0328157      0.631364      0.209868      0.228808    0.00576399    0.00127118    0.00811958       1.12477       Opttag_D
      25       46.6627       2.04537       1.00000  0.000000E+00       1.00000  0.000000E+00      0.794011       64.4474       1.00000       24.8894  0.000000E+00      0.978627  0.000000E+00      0.978627  0.000000E+00  0.000000E+00      -1.95725       46.8739       1.38831        1       Scatag_D
      25       1858.75       6904.73  0.000000E+00  0.000000E+00       2253.26  0.000000E+00       3886.93       6256.87  0.000000E+00       11134.3       64.3986       10445.4 -1.000000E+10 -1.000000E+10 -1.000000E+10 -1.000000E+10 -1.000000E+10  0.000000E+00  0.000000E+00  0.000000E+00  0.000000E+00  0.000000E+00  0.000000E+00  0.000000E+00  0.000000E+00  0.000000E+00  0.000000E+00  0.000000E+00  0.000000E+00       Funtag_D
      26  5.412148E+05  4.984311E+05       42783.7       7.26717     0.0332882      0.641409      0.209867      0.228789    0.00576147    0.00130915    0.00810478       1.12484       Opttag_D
      26       46.6661       2.04586       1.00000  0.000000E+00       1.00000  0.000000E+00      0.794014       64.4533       1.00000       24.8885  0.000000E+00      0.978391  0.000000E+00      0.978391  0.000000E+00  0.000000E+00      -1.95678       46.8739       1.38831        1       Scatag_D
      26       1851.98       6905.51  0.000000E+00  0.000000E+00       2253.91  0.000000E+00       3882.21       6248.91  0.000000E+00       11135.5       64.7179       10441.0 -1.000000E+10 -1.000000E+10 -1.000000E+10 -1.000000E+10 -1.000000E+10  0.000000E+00  0.000000E+00  0.000000E+00  0.000000E+00  0.000000E+00  0.000000E+00  0.000000E+00  0.000000E+00  0.000000E+00  0.000000E+00  0.000000E+00  0.000000E+00       Funtag_D
      27  5.411939E+05  4.984234E+05       42770.5       8.48218     0.0342992      0.662534      0.209856      0.228763    0.00577859    0.00133238    0.00809346       1.12513       Opttag_D
      27       46.6727       2.04653       1.00000  0.000000E+00       1.00000  0.000000E+00      0.794013       64.4658       1.00000       24.8853  0.000000E+00      0.977566  0.000000E+00      0.977566  0.000000E+00  0.000000E+00      -1.95513       46.8739       1.38831        1       Scatag_D
      27       1846.80       6909.11  0.000000E+00  0.000000E+00       2255.17  0.000000E+00       3877.44       6240.20  0.000000E+00       11138.6       65.0645       10438.1 -1.000000E+10 -1.000000E+10 -1.000000E+10 -1.000000E+10 -1.000000E+10  0.000000E+00  0.000000E+00  0.000000E+00  0.000000E+00  0.000000E+00  0.000000E+00  0.000000E+00  0.000000E+00  0.000000E+00  0.000000E+00  0.000000E+00  0.000000E+00       Funtag_D
      28  5.411768E+05  4.983984E+05       42778.3       15.0873     0.0364864      0.706733      0.209827      0.228719    0.00583377    0.00132867    0.00808483       1.12584       Opttag_D
      28       46.6863       2.04760       1.00000  0.000000E+00       1.00000  0.000000E+00      0.794012       64.4903       1.00000       24.8803  0.000000E+00      0.975681  0.000000E+00      0.975681  0.000000E+00  0.000000E+00      -1.95136       46.8739       1.38831        1       Scatag_D
      28       1842.87       6917.76  0.000000E+00  0.000000E+00       2257.69  0.000000E+00       3871.78       6239.84  0.000000E+00       11145.2       65.4976       10437.7 -1.000000E+10 -1.000000E+10 -1.000000E+10 -1.000000E+10 -1.000000E+10  0.000000E+00  0.000000E+00  0.000000E+00  0.000000E+00  0.000000E+00  0.000000E+00  0.000000E+00  0.000000E+00  0.000000E+00  0.000000E+00  0.000000E+00  0.000000E+00       Funtag_D
      29  5.411510E+05  4.983883E+05       42762.7       7.52594     0.0368672      0.714276      0.209818      0.228713    0.00585633    0.00131059    0.00808416       1.12577       Opttag_D
      29       46.6884       2.04762       1.00000  0.000000E+00       1.00000  0.000000E+00      0.794011       64.4949       1.00000       24.8792  0.000000E+00      0.975212  0.000000E+00      0.975212  0.000000E+00  0.000000E+00      -1.95042       46.8739       1.38831        1       Scatag_D
      29       1842.56       6916.92  0.000000E+00  0.000000E+00       2258.04  0.000000E+00       3872.63       6224.57  0.000000E+00       11145.5       65.3830       10437.1 -1.000000E+10 -1.000000E+10 -1.000000E+10 -1.000000E+10 -1.000000E+10  0.000000E+00  0.000000E+00  0.000000E+00  0.000000E+00  0.000000E+00  0.000000E+00  0.000000E+00  0.000000E+00  0.000000E+00  0.000000E+00  0.000000E+00  0.000000E+00       Funtag_D
      30  5.411344E+05  4.983703E+05       42764.1       6.80299     0.0373979      0.724601      0.209806      0.228704    0.00589600    0.00127777    0.00808591       1.12571       Opttag_D
      30       46.6912       2.04750       1.00000  0.000000E+00       1.00000  0.000000E+00      0.794010       64.5006       1.00000       24.8784  0.000000E+00      0.974497  0.000000E+00      0.974497  0.000000E+00  0.000000E+00      -1.94899       46.8739       1.38831        1       Scatag_D
      30       1843.36       6916.18  0.000000E+00  0.000000E+00       2258.49  0.000000E+00       3874.62       6224.58  0.000000E+00       11145.3       65.1517       10436.4 -1.000000E+10 -1.000000E+10 -1.000000E+10 -1.000000E+10 -1.000000E+10  0.000000E+00  0.000000E+00  0.000000E+00  0.000000E+00  0.000000E+00  0.000000E+00  0.000000E+00  0.000000E+00  0.000000E+00  0.000000E+00  0.000000E+00  0.000000E+00       Funtag_D
      31  5.411156E+05  4.983422E+05       42773.4       7.61386     0.0385037      0.745411      0.209785      0.228684    0.00595824    0.00123478    0.00809053       1.12586       Opttag_D
      31       46.6973       2.04750       1.00000  0.000000E+00       1.00000  0.000000E+00      0.794010       64.5118       1.00000       24.8778  0.000000E+00      0.973267  0.000000E+00      0.973267  0.000000E+00  0.000000E+00      -1.94653       46.8739       1.38831        1       Scatag_D
      31       1845.47       6918.01  0.000000E+00  0.000000E+00       2259.49  0.000000E+00       3877.04       6226.64  0.000000E+00       11145.8       64.9052       10436.1 -1.000000E+10 -1.000000E+10 -1.000000E+10 -1.000000E+10 -1.000000E+10  0.000000E+00  0.000000E+00  0.000000E+00  0.000000E+00  0.000000E+00  0.000000E+00  0.000000E+00  0.000000E+00  0.000000E+00  0.000000E+00  0.000000E+00  0.000000E+00       Funtag_D
      32  5.410994E+05  4.982921E+05       42807.3       16.3640     0.0407761      0.785167      0.209752      0.228643    0.00606941    0.00116771    0.00809945       1.12640       Opttag_D
      32       46.7089       2.04757       1.00000  0.000000E+00       1.00000  0.000000E+00      0.794016       64.5331       1.00000       24.8800  0.000000E+00      0.971092  0.000000E+00      0.971092  0.000000E+00  0.000000E+00      -1.94218       46.8739       1.38831        1       Scatag_D
      32       1849.54       6924.69  0.000000E+00  0.000000E+00       2261.39  0.000000E+00       3881.87       6245.76  0.000000E+00       11145.7       64.5660       10433.8 -1.000000E+10 -1.000000E+10 -1.000000E+10 -1.000000E+10 -1.000000E+10  0.000000E+00  0.000000E+00  0.000000E+00  0.000000E+00  0.000000E+00  0.000000E+00  0.000000E+00  0.000000E+00  0.000000E+00  0.000000E+00  0.000000E+00  0.000000E+00       Funtag_D
      33  5.410716E+05  4.982767E+05       42794.9       7.67805     0.0417389      0.801108      0.209741      0.228631    0.00610334    0.00113597    0.00810114       1.12659       Opttag_D
      33       46.7136       2.04775       1.00000  0.000000E+00       1.00000  0.000000E+00      0.794021       64.5419       1.00000       24.8814  0.000000E+00      0.970268  0.000000E+00      0.970268  0.000000E+00  0.000000E+00      -1.94054       46.8739       1.38831        1       Scatag_D
      33       1850.31       6926.99  0.000000E+00  0.000000E+00       2262.22  0.000000E+00       3883.28       6228.44  0.000000E+00       11146.2       64.5218       10432.9 -1.000000E+10 -1.000000E+10 -1.000000E+10 -1.000000E+10 -1.000000E+10  0.000000E+00  0.000000E+00  0.000000E+00  0.000000E+00  0.000000E+00  0.000000E+00  0.000000E+00  0.000000E+00  0.000000E+00  0.000000E+00  0.000000E+00  0.000000E+00       Funtag_D
      34  5.410561E+05  4.982697E+05       42786.4       6.30769     0.0423353      0.809630      0.209737      0.228622    0.00611875    0.00112723    0.00809710       1.12665       Opttag_D
      34       46.7162       2.04788       1.00000  0.000000E+00       1.00000  0.000000E+00      0.794026       64.5472       1.00000       24.8832  0.000000E+00      0.969949  0.000000E+00      0.969949  0.000000E+00  0.000000E+00      -1.93990       46.8739       1.38831        1       Scatag_D
      34       1848.47       6927.75  0.000000E+00  0.000000E+00       2262.65  0.000000E+00       3883.47       6224.70  0.000000E+00       11145.5       64.5939       10429.3 -1.000000E+10 -1.000000E+10 -1.000000E+10 -1.000000E+10 -1.000000E+10  0.000000E+00  0.000000E+00  0.000000E+00  0.000000E+00  0.000000E+00  0.000000E+00  0.000000E+00  0.000000E+00  0.000000E+00  0.000000E+00  0.000000E+00  0.000000E+00       Funtag_D
      35  5.410385E+05  4.982593E+05       42779.2       7.40879     0.0434212      0.824330      0.209729      0.228611    0.00614191    0.00109693    0.00809453       1.12680       Opttag_D
      35       46.7204       2.04814       1.00000  0.000000E+00       1.00000  0.000000E+00      0.794035       64.5575       1.00000       24.8862  0.000000E+00      0.969389  0.000000E+00      0.969389  0.000000E+00  0.000000E+00      -1.93878       46.8739       1.38831        1       Scatag_D
      35       1847.30       6929.57  0.000000E+00  0.000000E+00       2263.39  0.000000E+00       3884.42       6221.33  0.000000E+00       11144.3       64.7104       10424.2 -1.000000E+10 -1.000000E+10 -1.000000E+10 -1.000000E+10 -1.000000E+10  0.000000E+00  0.000000E+00  0.000000E+00  0.000000E+00  0.000000E+00  0.000000E+00  0.000000E+00  0.000000E+00  0.000000E+00  0.000000E+00  0.000000E+00  0.000000E+00       Funtag_D
      36  5.410160E+05  4.982490E+05       42767.0       12.1004     0.0453687      0.847413      0.209721      0.228604    0.00616467    0.00101297    0.00809056       1.12682       Opttag_D
      36       46.7270       2.04866       1.00000  0.000000E+00       1.00000  0.000000E+00      0.794052       64.5758       1.00000       24.8919  0.000000E+00      0.968816  0.000000E+00      0.968816  0.000000E+00  0.000000E+00      -1.93763       46.8739       1.38831        1       Scatag_D
      36       1845.48       6929.89  0.000000E+00  0.000000E+00       2264.62  0.000000E+00       3887.12       6215.79  0.000000E+00       11142.7       64.7927       10416.5 -1.000000E+10 -1.000000E+10 -1.000000E+10 -1.000000E+10 -1.000000E+10  0.000000E+00  0.000000E+00  0.000000E+00  0.000000E+00  0.000000E+00  0.000000E+00  0.000000E+00  0.000000E+00  0.000000E+00  0.000000E+00  0.000000E+00  0.000000E+00       Funtag_D
      37  5.409926E+05  4.982429E+05       42749.7       7.92465     0.0473133      0.868884      0.209715      0.228605    0.00617826  9.011106E-04    0.00809200       1.12690       Opttag_D
      37       46.7331       2.04924       1.00000  0.000000E+00       1.00000  0.000000E+00      0.794068       64.5936       1.00000       24.8985  0.000000E+00      0.968140  0.000000E+00      0.968140  0.000000E+00  0.000000E+00      -1.93628       46.8739       1.38831        1       Scatag_D
      37       1846.14       6930.86  0.000000E+00  0.000000E+00       2265.89  0.000000E+00       3890.50       6196.58  0.000000E+00       11142.4       64.8069       10412.6 -1.000000E+10 -1.000000E+10 -1.000000E+10 -1.000000E+10 -1.000000E+10  0.000000E+00  0.000000E+00  0.000000E+00  0.000000E+00  0.000000E+00  0.000000E+00  0.000000E+00  0.000000E+00  0.000000E+00  0.000000E+00  0.000000E+00  0.000000E+00       Funtag_D
      38  5.409776E+05  4.982433E+05       42734.3       6.28245     0.0477791      0.871340      0.209714      0.228615    0.00617726  8.577558E-04    0.00808800       1.12664       Opttag_D
      38       46.7336       2.04928       1.00000  0.000000E+00       1.00000  0.000000E+00      0.794070       64.5975       1.00000       24.9009  0.000000E+00      0.968137  0.000000E+00      0.968137  0.000000E+00  0.000000E+00      -1.93627       46.8739       1.38831        1       Scatag_D
      38       1844.31       6927.60  0.000000E+00  0.000000E+00       2266.08  0.000000E+00       3891.58       6188.43  0.000000E+00       11141.6       64.6782       10410.0 -1.000000E+10 -1.000000E+10 -1.000000E+10 -1.000000E+10 -1.000000E+10  0.000000E+00  0.000000E+00  0.000000E+00  0.000000E+00  0.000000E+00  0.000000E+00  0.000000E+00  0.000000E+00  0.000000E+00  0.000000E+00  0.000000E+00  0.000000E+00       Funtag_D
      39  5.409559E+05  4.982426E+05       42713.3       7.73851     0.0488300      0.875464      0.209716      0.228639    0.00617887  7.575126E-04    0.00808312       1.12614       Opttag_D
      39       46.7343       2.04923       1.00000  0.000000E+00       1.00000  0.000000E+00      0.794076       64.6040       1.00000       24.9077  0.000000E+00      0.968206  0.000000E+00      0.968206  0.000000E+00  0.000000E+00      -1.93641       46.8739       1.38831        1       Scatag_D
      39       1842.09       6921.47  0.000000E+00  0.000000E+00       2266.50  0.000000E+00       3894.66       6177.92  0.000000E+00       11140.3       64.3443       10406.0 -1.000000E+10 -1.000000E+10 -1.000000E+10 -1.000000E+10 -1.000000E+10  0.000000E+00  0.000000E+00  0.000000E+00  0.000000E+00  0.000000E+00  0.000000E+00  0.000000E+00  0.000000E+00  0.000000E+00  0.000000E+00  0.000000E+00  0.000000E+00       Funtag_D
      40  5.409430E+05  4.982354E+05       42707.6       11.2171     0.0504237      0.884159      0.209716      0.228663    0.00619478  6.388256E-04    0.00808181       1.12588       Opttag_D
      40       46.7368       2.04922       1.00000  0.000000E+00       1.00000  0.000000E+00      0.794086       64.6149       1.00000       24.9180  0.000000E+00      0.967750  0.000000E+00      0.967750  0.000000E+00  0.000000E+00      -1.93550       46.8739       1.38831        1       Scatag_D
      40       1841.49       6918.29  0.000000E+00  0.000000E+00       2267.39  0.000000E+00       3899.29       6173.20  0.000000E+00       11140.2       64.0615       10403.7 -1.000000E+10 -1.000000E+10 -1.000000E+10 -1.000000E+10 -1.000000E+10  0.000000E+00  0.000000E+00  0.000000E+00  0.000000E+00  0.000000E+00  0.000000E+00  0.000000E+00  0.000000E+00  0.000000E+00  0.000000E+00  0.000000E+00  0.000000E+00       Funtag_D
      41  5.409264E+05  4.982299E+05       42696.5       7.20171     0.0507439      0.884763      0.209715      0.228668    0.00620706  6.270887E-04    0.00807830       1.12580       Opttag_D
      41       46.7370       2.04904       1.00000  0.000000E+00       1.00000  0.000000E+00      0.794088       64.6151       1.00000       24.9211  0.000000E+00      0.967643  0.000000E+00      0.967643  0.000000E+00  0.000000E+00      -1.93529       46.8739       1.38831        1       Scatag_D
      41       1839.89       6917.33  0.000000E+00  0.000000E+00       2267.56  0.000000E+00       3900.25       6165.19  0.000000E+00       11140.1       64.0024       10402.2 -1.000000E+10 -1.000000E+10 -1.000000E+10 -1.000000E+10 -1.000000E+10  0.000000E+00  0.000000E+00  0.000000E+00  0.000000E+00  0.000000E+00  0.000000E+00  0.000000E+00  0.000000E+00  0.000000E+00  0.000000E+00  0.000000E+00  0.000000E+00       Funtag_D
      42  5.409048E+05  4.982128E+05       42692.0       6.46532     0.0519210      0.890113      0.209710      0.228682    0.00624482  5.820571E-04    0.00807393       1.12581       Opttag_D
      42       46.7389       2.04867       1.00000  0.000000E+00       1.00000  0.000000E+00      0.794100       64.6193       1.00000       24.9303  0.000000E+00      0.967030  0.000000E+00      0.967030  0.000000E+00  0.000000E+00      -1.93406       46.8739       1.38831        1       Scatag_D
      42       1837.90       6917.46  0.000000E+00  0.000000E+00       2268.28  0.000000E+00       3903.39       6161.11  0.000000E+00       11140.7       63.9450       10399.2 -1.000000E+10 -1.000000E+10 -1.000000E+10 -1.000000E+10 -1.000000E+10  0.000000E+00  0.000000E+00  0.000000E+00  0.000000E+00  0.000000E+00  0.000000E+00  0.000000E+00  0.000000E+00  0.000000E+00  0.000000E+00  0.000000E+00  0.000000E+00       Funtag_D
      43  5.408896E+05  4.981963E+05       42693.2       7.10384     0.0531207      0.895842      0.209704      0.228698    0.00628138  5.316361E-04    0.00807261       1.12587       Opttag_D
      43       46.7410       2.04838       1.00000  0.000000E+00       1.00000  0.000000E+00      0.794113       64.6252       1.00000       24.9382  0.000000E+00      0.966424  0.000000E+00      0.966424  0.000000E+00  0.000000E+00      -1.93285       46.8739       1.38831        1       Scatag_D
      43       1837.30       6918.24  0.000000E+00  0.000000E+00       2269.01  0.000000E+00       3906.49       6159.37  0.000000E+00       11142.3       63.9351       10396.5 -1.000000E+10 -1.000000E+10 -1.000000E+10 -1.000000E+10 -1.000000E+10  0.000000E+00  0.000000E+00  0.000000E+00  0.000000E+00  0.000000E+00  0.000000E+00  0.000000E+00  0.000000E+00  0.000000E+00  0.000000E+00  0.000000E+00  0.000000E+00       Funtag_D
      44  5.408742E+05  4.981444E+05       42729.8       14.7451     0.0566011      0.912073      0.209683      0.228749    0.00639638  4.163026E-04    0.00807866       1.12642       Opttag_D
      44       46.7470       2.04745       1.00000  0.000000E+00       1.00000  0.000000E+00      0.794161       64.6416       1.00000       24.9602  0.000000E+00      0.964569  0.000000E+00      0.964569  0.000000E+00  0.000000E+00      -1.92914       46.8739       1.38831        1       Scatag_D
      44       1840.06       6924.92  0.000000E+00  0.000000E+00       2271.11  0.000000E+00       3915.29       6172.65  0.000000E+00       11150.6       64.0654       10391.2 -1.000000E+10 -1.000000E+10 -1.000000E+10 -1.000000E+10 -1.000000E+10  0.000000E+00  0.000000E+00  0.000000E+00  0.000000E+00  0.000000E+00  0.000000E+00  0.000000E+00  0.000000E+00  0.000000E+00  0.000000E+00  0.000000E+00  0.000000E+00       Funtag_D
      45  5.408481E+05  4.981313E+05       42716.9       6.74753     0.0570367      0.914524      0.209674      0.228753    0.00642538  4.081586E-04    0.00807893       1.12646       Opttag_D
      45       46.7477       2.04718       1.00000  0.000000E+00       1.00000  0.000000E+00      0.794169       64.6443       1.00000       24.9612  0.000000E+00      0.964514  0.000000E+00      0.964514  0.000000E+00  0.000000E+00      -1.92903       46.8739       1.38831        1       Scatag_D
      45       1840.18       6925.40  0.000000E+00  0.000000E+00       2271.31  0.000000E+00       3915.50       6159.31  0.000000E+00       11152.3       64.1241       10388.8 -1.000000E+10 -1.000000E+10 -1.000000E+10 -1.000000E+10 -1.000000E+10  0.000000E+00  0.000000E+00  0.000000E+00  0.000000E+00  0.000000E+00  0.000000E+00  0.000000E+00  0.000000E+00  0.000000E+00  0.000000E+00  0.000000E+00  0.000000E+00       Funtag_D
      46  5.408366E+05  4.981201E+05       42716.5       5.97654     0.0570511      0.915024      0.209665      0.228749    0.00645015  4.285186E-04    0.00807971       1.12645       Opttag_D
      46       46.7477       2.04693       1.00000  0.000000E+00       1.00000  0.000000E+00      0.794173       64.6448       1.00000       24.9594  0.000000E+00      0.964514  0.000000E+00      0.964514  0.000000E+00  0.000000E+00      -1.92903       46.8739       1.38831        1       Scatag_D
      46       1840.54       6925.35  0.000000E+00  0.000000E+00       2271.22  0.000000E+00       3914.35       6161.41  0.000000E+00       11152.8       64.1741       10386.7 -1.000000E+10 -1.000000E+10 -1.000000E+10 -1.000000E+10 -1.000000E+10  0.000000E+00  0.000000E+00  0.000000E+00  0.000000E+00  0.000000E+00  0.000000E+00  0.000000E+00  0.000000E+00  0.000000E+00  0.000000E+00  0.000000E+00  0.000000E+00       Funtag_D
      47  5.408165E+05  4.980802E+05       42736.2       7.34629     0.0580422      0.921156      0.209640      0.228750    0.00653856  4.438485E-04    0.00809106       1.12674       Opttag_D
      47       46.7494       2.04607       1.00000  0.000000E+00       1.00000  0.000000E+00      0.794197       64.6493       1.00000       24.9612  0.000000E+00      0.964221  0.000000E+00      0.964221  0.000000E+00  0.000000E+00      -1.92844       46.8739       1.38831        1       Scatag_D
      47       1845.71       6928.91  0.000000E+00  0.000000E+00       2271.60  0.000000E+00       3913.74       6171.74  0.000000E+00       11157.2       64.2996       10383.0 -1.000000E+10 -1.000000E+10 -1.000000E+10 -1.000000E+10 -1.000000E+10  0.000000E+00  0.000000E+00  0.000000E+00  0.000000E+00  0.000000E+00  0.000000E+00  0.000000E+00  0.000000E+00  0.000000E+00  0.000000E+00  0.000000E+00  0.000000E+00       Funtag_D
      48  5.408062E+05  4.980068E+05       42799.4       16.0217     0.0604339      0.957880      0.209598      0.228763    0.00670109  4.554657E-04    0.00811734       1.12753       Opttag_D
      48       46.7533       2.04456       1.00000  0.000000E+00       1.00000  0.000000E+00      0.794248       64.6594       1.00000       24.9697  0.000000E+00      0.963348  0.000000E+00      0.963348  0.000000E+00  0.000000E+00      -1.92670       46.8739       1.38831        1       Scatag_D
      48       1857.72       6938.59  0.000000E+00  0.000000E+00       2272.80  0.000000E+00       3913.60       6205.76  0.000000E+00       11166.8       64.4472       10379.6 -1.000000E+10 -1.000000E+10 -1.000000E+10 -1.000000E+10 -1.000000E+10  0.000000E+00  0.000000E+00  0.000000E+00  0.000000E+00  0.000000E+00  0.000000E+00  0.000000E+00  0.000000E+00  0.000000E+00  0.000000E+00  0.000000E+00  0.000000E+00       Funtag_D
      49  5.407822E+05  4.979933E+05       42788.9       7.54212     0.0610212      0.967946      0.209589      0.228769    0.00673100  4.440880E-04    0.00811898       1.12766       Opttag_D
      49       46.7543       2.04428       1.00000  0.000000E+00       1.00000  0.000000E+00      0.794260       64.6613       1.00000       24.9726  0.000000E+00      0.963217  0.000000E+00      0.963217  0.000000E+00  0.000000E+00      -1.92643       46.8739       1.38831        1       Scatag_D
      49       1858.47       6940.24  0.000000E+00  0.000000E+00       2273.12  0.000000E+00       3913.68       6191.52  0.000000E+00       11168.7       64.4379       10378.7 -1.000000E+10 -1.000000E+10 -1.000000E+10 -1.000000E+10 -1.000000E+10  0.000000E+00  0.000000E+00  0.000000E+00  0.000000E+00  0.000000E+00  0.000000E+00  0.000000E+00  0.000000E+00  0.000000E+00  0.000000E+00  0.000000E+00  0.000000E+00       Funtag_D
      50  5.407677E+05  4.979715E+05       42796.2       5.76336     0.0620978      0.985961      0.209579      0.228781    0.00677923  4.279198E-04    0.00812170       1.12793       Opttag_D
      50       46.7560       2.04385       1.00000  0.000000E+00       1.00000  0.000000E+00      0.794280       64.6649       1.00000       24.9785  0.000000E+00      0.962894  0.000000E+00      0.962894  0.000000E+00  0.000000E+00      -1.92579       46.8739       1.38831        1       Scatag_D
      50       1859.71       6943.47  0.000000E+00  0.000000E+00       2273.77  0.000000E+00       3913.72       6192.63  0.000000E+00       11171.8       64.4125       10376.7 -1.000000E+10 -1.000000E+10 -1.000000E+10 -1.000000E+10 -1.000000E+10  0.000000E+00  0.000000E+00  0.000000E+00  0.000000E+00  0.000000E+00  0.000000E+00  0.000000E+00  0.000000E+00  0.000000E+00  0.000000E+00  0.000000E+00  0.000000E+00       Funtag_D
      51  5.407534E+05  4.979391E+05       42814.3       6.34839     0.0637011       1.01159      0.209563      0.228802    0.00685093  4.080388E-04    0.00812767       1.12841       Opttag_D
      51       46.7586       2.04324       1.00000  0.000000E+00       1.00000  0.000000E+00      0.794309       64.6707       1.00000       24.9866  0.000000E+00      0.962400  0.000000E+00      0.962400  0.000000E+00  0.000000E+00      -1.92480       46.8739       1.38831        1       Scatag_D
      51       1862.45       6949.49  0.000000E+00  0.000000E+00       2274.73  0.000000E+00       3913.45       6199.13  0.000000E+00       11176.5       64.4238       10374.1 -1.000000E+10 -1.000000E+10 -1.000000E+10 -1.000000E+10 -1.000000E+10  0.000000E+00  0.000000E+00  0.000000E+00  0.000000E+00  0.000000E+00  0.000000E+00  0.000000E+00  0.000000E+00  0.000000E+00  0.000000E+00  0.000000E+00  0.000000E+00       Funtag_D
      52  5.407342E+05  4.978985E+05       42835.7       10.4006     0.0662527       1.04949      0.209543      0.228860    0.00694095  3.646839E-04    0.00812927       1.12913       Opttag_D
      52       46.7624       2.04253       1.00000  0.000000E+00       1.00000  0.000000E+00      0.794355       64.6804       1.00000       24.9994  0.000000E+00      0.961639  0.000000E+00      0.961639  0.000000E+00  0.000000E+00      -1.92328       46.8739       1.38831        1       Scatag_D
      52       1863.19       6958.30  0.000000E+00  0.000000E+00       2276.23  0.000000E+00       3912.37       6211.44  0.000000E+00       11182.6       64.5366       10367.1 -1.000000E+10 -1.000000E+10 -1.000000E+10 -1.000000E+10 -1.000000E+10  0.000000E+00  0.000000E+00  0.000000E+00  0.000000E+00  0.000000E+00  0.000000E+00  0.000000E+00  0.000000E+00  0.000000E+00  0.000000E+00  0.000000E+00  0.000000E+00       Funtag_D
      53  5.407134E+05  4.978608E+05       42852.7       6.63120     0.0690036       1.08861      0.209529      0.228925    0.00702453  2.947413E-04    0.00813355       1.12995       Opttag_D
      53       46.7664       2.04190       1.00000  0.000000E+00       1.00000  0.000000E+00      0.794403       64.6910       1.00000       25.0136  0.000000E+00      0.961109  0.000000E+00      0.961109  0.000000E+00  0.000000E+00      -1.92222       46.8739       1.38831        1       Scatag_D
      53       1865.15       6968.37  0.000000E+00  0.000000E+00       2277.84  0.000000E+00       3911.25       6215.46  0.000000E+00       11189.7       64.7187       10360.2 -1.000000E+10 -1.000000E+10 -1.000000E+10 -1.000000E+10 -1.000000E+10  0.000000E+00  0.000000E+00  0.000000E+00  0.000000E+00  0.000000E+00  0.000000E+00  0.000000E+00  0.000000E+00  0.000000E+00  0.000000E+00  0.000000E+00  0.000000E+00       Funtag_D
      54  5.406981E+05  4.978585E+05       42839.6       5.76712     0.0696252       1.09555      0.209529      0.228950    0.00702947  2.674349E-04    0.00812634       1.12995       Opttag_D
      54       46.7671       2.04193       1.00000  0.000000E+00       1.00000  0.000000E+00      0.794411       64.6945       1.00000       25.0158  0.000000E+00      0.961133  0.000000E+00      0.961133  0.000000E+00  0.000000E+00      -1.92227       46.8739       1.38831        1       Scatag_D
      54       1861.84       6968.45  0.000000E+00  0.000000E+00       2278.10  0.000000E+00       3909.64       6214.04  0.000000E+00       11188.8       64.8457       10353.9 -1.000000E+10 -1.000000E+10 -1.000000E+10 -1.000000E+10 -1.000000E+10  0.000000E+00  0.000000E+00  0.000000E+00  0.000000E+00  0.000000E+00  0.000000E+00  0.000000E+00  0.000000E+00  0.000000E+00  0.000000E+00  0.000000E+00  0.000000E+00       Funtag_D
      55  5.406725E+05  4.978600E+05       42812.5       8.39758     0.0711555       1.11173      0.209531      0.229015    0.00702635  1.869529E-04    0.00811541       1.12995       Opttag_D
      55       46.7687       2.04221       1.00000  0.000000E+00       1.00000  0.000000E+00      0.794430       64.7040       1.00000       25.0204  0.000000E+00      0.961505  0.000000E+00      0.961505  0.000000E+00  0.000000E+00      -1.92301       46.8739       1.38831        1       Scatag_D
      55       1856.84       6968.44  0.000000E+00  0.000000E+00       2278.54  0.000000E+00       3905.09       6214.07  0.000000E+00       11185.8       65.1715       10338.6 -1.000000E+10 -1.000000E+10 -1.000000E+10 -1.000000E+10 -1.000000E+10  0.000000E+00  0.000000E+00  0.000000E+00  0.000000E+00  0.000000E+00  0.000000E+00  0.000000E+00  0.000000E+00  0.000000E+00  0.000000E+00  0.000000E+00  0.000000E+00       Funtag_D
      56  5.406613E+05  4.978583E+05       42803.0       13.2814     0.0734540       1.13776      0.209534      0.229093    0.00702987  5.569065E-05    0.00811260       1.13035       Opttag_D
      56       46.7712       2.04246       1.00000  0.000000E+00       1.00000  0.000000E+00      0.794466       64.7151       1.00000       25.0293  0.000000E+00      0.961854  0.000000E+00      0.961854  0.000000E+00  0.000000E+00      -1.92371       46.8739       1.38831        1       Scatag_D
      56       1855.55       6973.31  0.000000E+00  0.000000E+00       2279.34  0.000000E+00       3899.96       6220.40  0.000000E+00       11185.2       65.3986       10323.9 -1.000000E+10 -1.000000E+10 -1.000000E+10 -1.000000E+10 -1.000000E+10  0.000000E+00  0.000000E+00  0.000000E+00  0.000000E+00  0.000000E+00  0.000000E+00  0.000000E+00  0.000000E+00  0.000000E+00  0.000000E+00  0.000000E+00  0.000000E+00       Funtag_D
      57  5.406422E+05  4.978621E+05       42780.1       7.24470     0.0732421       1.13430      0.209534      0.229085    0.00702151  6.706831E-05    0.00811040       1.13005       Opttag_D
      57       46.7707       2.04254       1.00000  0.000000E+00       1.00000  0.000000E+00      0.794462       64.7145       1.00000       25.0275  0.000000E+00      0.962054  0.000000E+00      0.962054  0.000000E+00  0.000000E+00      -1.92411       46.8739       1.38831        1       Scatag_D
      57       1854.55       6969.64  0.000000E+00  0.000000E+00       2279.07  0.000000E+00       3898.74       6207.70  0.000000E+00       11183.0       65.3122       10322.0 -1.000000E+10 -1.000000E+10 -1.000000E+10 -1.000000E+10 -1.000000E+10  0.000000E+00  0.000000E+00  0.000000E+00  0.000000E+00  0.000000E+00  0.000000E+00  0.000000E+00  0.000000E+00  0.000000E+00  0.000000E+00  0.000000E+00  0.000000E+00       Funtag_D
      58  5.406234E+05  4.978643E+05       42759.1       5.95945     0.0738649       1.14030      0.209539      0.229099    0.00701668  3.101909E-05    0.00811035       1.12987       Opttag_D
      58       46.7710       2.04267       1.00000  0.000000E+00       1.00000  0.000000E+00      0.794470       64.7170       1.00000       25.0291  0.000000E+00      0.962389  0.000000E+00      0.962389  0.000000E+00  0.000000E+00      -1.92478       46.8739       1.38831        1       Scatag_D
      58       1854.52       6967.41  0.000000E+00  0.000000E+00       2279.02  0.000000E+00       3895.06       6202.39  0.000000E+00       11180.6       65.1596       10315.0 -1.000000E+10 -1.000000E+10 -1.000000E+10 -1.000000E+10 -1.000000E+10  0.000000E+00  0.000000E+00  0.000000E+00  0.000000E+00  0.000000E+00  0.000000E+00  0.000000E+00  0.000000E+00  0.000000E+00  0.000000E+00  0.000000E+00  0.000000E+00       Funtag_D
      59  5.406076E+05  4.978665E+05       42741.1       6.87228     0.0751195       1.15281      0.209543      0.229137    0.00701174 -4.742688E-05    0.00811229       1.12996       Opttag_D
      59       46.7718       2.04284       1.00000  0.000000E+00       1.00000  0.000000E+00      0.794489       64.7215       1.00000       25.0336  0.000000E+00      0.962682  0.000000E+00      0.962682  0.000000E+00  0.000000E+00      -1.92536       46.8739       1.38831        1       Scatag_D
      59       1855.41       6968.52  0.000000E+00  0.000000E+00       2279.25  0.000000E+00       3890.22       6199.14  0.000000E+00       11178.2       64.9977       10305.3 -1.000000E+10 -1.000000E+10 -1.000000E+10 -1.000000E+10 -1.000000E+10  0.000000E+00  0.000000E+00  0.000000E+00  0.000000E+00  0.000000E+00  0.000000E+00  0.000000E+00  0.000000E+00  0.000000E+00  0.000000E+00  0.000000E+00  0.000000E+00       Funtag_D
      60  5.405811E+05  4.978769E+05       42704.3       12.1071     0.0778373       1.17767      0.209560      0.229241    0.00698889 -2.470749E-04    0.00811374       1.13024       Opttag_D
      60       46.7732       2.04325       1.00000  0.000000E+00       1.00000  0.000000E+00      0.794531       64.7283       1.00000       25.0447  0.000000E+00      0.963084  0.000000E+00      0.963084  0.000000E+00  0.000000E+00      -1.92617       46.8739       1.38831        1       Scatag_D
      60       1856.07       6972.04  0.000000E+00  0.000000E+00       2279.82  0.000000E+00       3880.67       6197.44  0.000000E+00       11171.9       64.6419       10281.7 -1.000000E+10 -1.000000E+10 -1.000000E+10 -1.000000E+10 -1.000000E+10  0.000000E+00  0.000000E+00  0.000000E+00  0.000000E+00  0.000000E+00  0.000000E+00  0.000000E+00  0.000000E+00  0.000000E+00  0.000000E+00  0.000000E+00  0.000000E+00       Funtag_D
      61  5.405533E+05  4.978969E+05       42656.4       9.52450     0.0811966       1.20717      0.209583      0.229383    0.00694458 -5.086413E-04    0.00811945       1.13075       Opttag_D
      61       46.7751       2.04394       1.00000  0.000000E+00       1.00000  0.000000E+00      0.794586       64.7358       1.00000       25.0599  0.000000E+00      0.963452  0.000000E+00      0.963452  0.000000E+00  0.000000E+00      -1.92690       46.8739       1.38831        1       Scatag_D
      61       1858.69       6978.29  0.000000E+00  0.000000E+00       2280.75  0.000000E+00       3871.32       6183.37  0.000000E+00       11166.4       64.3341       10253.3 -1.000000E+10 -1.000000E+10 -1.000000E+10 -1.000000E+10 -1.000000E+10  0.000000E+00  0.000000E+00  0.000000E+00  0.000000E+00  0.000000E+00  0.000000E+00  0.000000E+00  0.000000E+00  0.000000E+00  0.000000E+00  0.000000E+00  0.000000E+00       Funtag_D
      62  5.405296E+05  4.979049E+05       42624.6       6.97076     0.0818745       1.21157      0.209591      0.229429    0.00692665 -5.917581E-04    0.00811002       1.13060       Opttag_D
      62       46.7753       2.04416       1.00000  0.000000E+00       1.00000  0.000000E+00      0.794597       64.7359       1.00000       25.0631  0.000000E+00      0.963375  0.000000E+00      0.963375  0.000000E+00  0.000000E+00      -1.92675       46.8739       1.38831        1       Scatag_D
      62       1854.37       6976.39  0.000000E+00  0.000000E+00       2281.00  0.000000E+00       3869.93       6176.42  0.000000E+00       11162.0       64.3225       10240.2 -1.000000E+10 -1.000000E+10 -1.000000E+10 -1.000000E+10 -1.000000E+10  0.000000E+00  0.000000E+00  0.000000E+00  0.000000E+00  0.000000E+00  0.000000E+00  0.000000E+00  0.000000E+00  0.000000E+00  0.000000E+00  0.000000E+00  0.000000E+00       Funtag_D
      63  5.404896E+05  4.979344E+05       42555.1       9.13306     0.0837646       1.23170      0.209613      0.229557    0.00686140 -8.233833E-04    0.00809320       1.13023       Opttag_D
      63       46.7763       2.04501       1.00000  0.000000E+00       1.00000  0.000000E+00      0.794630       64.7367       1.00000       25.0719  0.000000E+00      0.963386  0.000000E+00      0.963386  0.000000E+00  0.000000E+00      -1.92677       46.8739       1.38831        1       Scatag_D
      63       1846.68       6971.93  0.000000E+00  0.000000E+00       2281.71  0.000000E+00       3867.72       6169.63  0.000000E+00       11149.5       64.4102       10203.6 -1.000000E+10 -1.000000E+10 -1.000000E+10 -1.000000E+10 -1.000000E+10  0.000000E+00  0.000000E+00  0.000000E+00  0.000000E+00  0.000000E+00  0.000000E+00  0.000000E+00  0.000000E+00  0.000000E+00  0.000000E+00  0.000000E+00  0.000000E+00       Funtag_D
      64  5.404663E+05  4.979776E+05       42488.7       14.1591     0.0864406       1.29717      0.209648      0.229708    0.00676574   -0.00109070    0.00808510       1.12990       Opttag_D
      64       46.7784       2.04637       1.00000  0.000000E+00       1.00000  0.000000E+00      0.794683       64.7404       1.00000       25.0846  0.000000E+00      0.963696  0.000000E+00      0.963696  0.000000E+00  0.000000E+00      -1.92739       46.8739       1.38831        1       Scatag_D
      64       1842.99       6967.75  0.000000E+00  0.000000E+00       2282.70  0.000000E+00       3865.69       6167.87  0.000000E+00       11136.9       64.5044       10160.3 -1.000000E+10 -1.000000E+10 -1.000000E+10 -1.000000E+10 -1.000000E+10  0.000000E+00  0.000000E+00  0.000000E+00  0.000000E+00  0.000000E+00  0.000000E+00  0.000000E+00  0.000000E+00  0.000000E+00  0.000000E+00  0.000000E+00  0.000000E+00       Funtag_D
      65  5.404320E+05  4.979904E+05       42441.6       9.50075     0.0867772       1.30742      0.209655      0.229723    0.00673744   -0.00113693    0.00807234       1.12926       Opttag_D
      65       46.7791       2.04686       1.00000  0.000000E+00       1.00000  0.000000E+00      0.794692       64.7413       1.00000       25.0849  0.000000E+00      0.963873  0.000000E+00      0.963873  0.000000E+00  0.000000E+00      -1.92775       46.8739       1.38831        1       Scatag_D
      65       1837.18       6959.91  0.000000E+00  0.000000E+00       2282.84  0.000000E+00       3866.97       6153.50  0.000000E+00       11130.9       64.5981       10145.7 -1.000000E+10 -1.000000E+10 -1.000000E+10 -1.000000E+10 -1.000000E+10  0.000000E+00  0.000000E+00  0.000000E+00  0.000000E+00  0.000000E+00  0.000000E+00  0.000000E+00  0.000000E+00  0.000000E+00  0.000000E+00  0.000000E+00  0.000000E+00       Funtag_D
      66  5.403849E+05  4.980245E+05       42360.4       8.16099     0.0882014       1.34415      0.209670      0.229765    0.00666192   -0.00125310    0.00805629       1.12792       Opttag_D
      66       46.7814       2.04820       1.00000  0.000000E+00       1.00000  0.000000E+00      0.794729       64.7452       1.00000       25.0883  0.000000E+00      0.964433  0.000000E+00      0.964433  0.000000E+00  0.000000E+00      -1.92887       46.8739       1.38831        1       Scatag_D
      66       1829.88       6943.37  0.000000E+00  0.000000E+00       2283.32  0.000000E+00       3869.02       6139.16  0.000000E+00       11118.2       64.6521       10112.8 -1.000000E+10 -1.000000E+10 -1.000000E+10 -1.000000E+10 -1.000000E+10  0.000000E+00  0.000000E+00  0.000000E+00  0.000000E+00  0.000000E+00  0.000000E+00  0.000000E+00  0.000000E+00  0.000000E+00  0.000000E+00  0.000000E+00  0.000000E+00       Funtag_D
      67  5.403599E+05  4.980390E+05       42320.9       9.97296     0.0891652       1.36839      0.209681      0.229789    0.00662990   -0.00129885    0.00805225       1.12708       Opttag_D
      67       46.7826       2.04887       1.00000  0.000000E+00       1.00000  0.000000E+00      0.794754       64.7468       1.00000       25.0894  0.000000E+00      0.964482  0.000000E+00      0.964482  0.000000E+00  0.000000E+00      -1.92896       46.8739       1.38831        1       Scatag_D
      67       1828.04       6933.01  0.000000E+00  0.000000E+00       2283.52  0.000000E+00       3871.01       6135.61  0.000000E+00       11110.4       64.4835       10094.9 -1.000000E+10 -1.000000E+10 -1.000000E+10 -1.000000E+10 -1.000000E+10  0.000000E+00  0.000000E+00  0.000000E+00  0.000000E+00  0.000000E+00  0.000000E+00  0.000000E+00  0.000000E+00  0.000000E+00  0.000000E+00  0.000000E+00  0.000000E+00       Funtag_D
      68  5.403302E+05  4.980473E+05       42282.8       7.90022     0.0903234       1.39719      0.209685      0.229796    0.00661127   -0.00130220    0.00805664       1.12605       Opttag_D
      68       46.7840       2.04946       1.00000  0.000000E+00       1.00000  0.000000E+00      0.794789       64.7466       1.00000       25.0896  0.000000E+00      0.964361  0.000000E+00      0.964361  0.000000E+00  0.000000E+00      -1.92872       46.8739       1.38831        1       Scatag_D
      68       1830.04       6920.37  0.000000E+00  0.000000E+00       2283.69  0.000000E+00       3875.55       6128.92  0.000000E+00       11101.1       64.1976       10078.9 -1.000000E+10 -1.000000E+10 -1.000000E+10 -1.000000E+10 -1.000000E+10  0.000000E+00  0.000000E+00  0.000000E+00  0.000000E+00  0.000000E+00  0.000000E+00  0.000000E+00  0.000000E+00  0.000000E+00  0.000000E+00  0.000000E+00  0.000000E+00       Funtag_D
      69  5.403100E+05  4.980348E+05       42275.2       7.82173     0.0907059       1.40699      0.209674      0.229781    0.00663916   -0.00125298    0.00806132       1.12551       Opttag_D
      69       46.7843       2.04927       1.00000  0.000000E+00       1.00000  0.000000E+00      0.794805       64.7448       1.00000       25.0872  0.000000E+00      0.963987  0.000000E+00      0.963987  0.000000E+00  0.000000E+00      -1.92797       46.8739       1.38831        1       Scatag_D
      69       1832.17       6913.79  0.000000E+00  0.000000E+00       2283.60  0.000000E+00       3880.00       6132.05  0.000000E+00       11097.6       64.0106       10072.0 -1.000000E+10 -1.000000E+10 -1.000000E+10 -1.000000E+10 -1.000000E+10  0.000000E+00  0.000000E+00  0.000000E+00  0.000000E+00  0.000000E+00  0.000000E+00  0.000000E+00  0.000000E+00  0.000000E+00  0.000000E+00  0.000000E+00  0.000000E+00       Funtag_D
      70  5.402891E+05  4.980124E+05       42276.7       6.71493     0.0909752       1.41442      0.209654      0.229761    0.00668870   -0.00118459    0.00806623       1.12500       Opttag_D
      70       46.7844       2.04881       1.00000  0.000000E+00       1.00000  0.000000E+00      0.794819       64.7418       1.00000       25.0837  0.000000E+00      0.963476  0.000000E+00      0.963476  0.000000E+00  0.000000E+00      -1.92695       46.8739       1.38831        1       Scatag_D
      70       1834.40       6907.52  0.000000E+00  0.000000E+00       2283.43  0.000000E+00       3886.30       6140.67  0.000000E+00       11094.3       63.9284       10066.2 -1.000000E+10 -1.000000E+10 -1.000000E+10 -1.000000E+10 -1.000000E+10  0.000000E+00  0.000000E+00  0.000000E+00  0.000000E+00  0.000000E+00  0.000000E+00  0.000000E+00  0.000000E+00  0.000000E+00  0.000000E+00  0.000000E+00  0.000000E+00       Funtag_D
      71  5.402717E+05  4.979925E+05       42279.2       6.58438     0.0913093       1.42349      0.209635      0.229739    0.00673281   -0.00112771    0.00806722       1.12456       Opttag_D
      71       46.7848       2.04845       1.00000  0.000000E+00       1.00000  0.000000E+00      0.794837       64.7403       1.00000       25.0799  0.000000E+00      0.963121  0.000000E+00      0.963121  0.000000E+00  0.000000E+00      -1.92624       46.8739       1.38831        1       Scatag_D
      71       1834.85       6902.11  0.000000E+00  0.000000E+00       2283.28  0.000000E+00       3892.85       6150.49  0.000000E+00       11090.6       63.9928       10061.1 -1.000000E+10 -1.000000E+10 -1.000000E+10 -1.000000E+10 -1.000000E+10  0.000000E+00  0.000000E+00  0.000000E+00  0.000000E+00  0.000000E+00  0.000000E+00  0.000000E+00  0.000000E+00  0.000000E+00  0.000000E+00  0.000000E+00  0.000000E+00       Funtag_D
      72  5.402569E+05  4.979743E+05       42282.6       6.91150     0.0916685       1.43333      0.209615      0.229712    0.00677309   -0.00106914    0.00806575       1.12418       Opttag_D
      72       46.7854       2.04818       1.00000  0.000000E+00       1.00000  0.000000E+00      0.794856       64.7401       1.00000       25.0756  0.000000E+00      0.963244  0.000000E+00      0.963244  0.000000E+00  0.000000E+00      -1.92649       46.8739       1.38831        1       Scatag_D
      72       1834.18       6897.39  0.000000E+00  0.000000E+00       2283.20  0.000000E+00       3898.26       6161.54  0.000000E+00       11086.8       64.1845       10057.1 -1.000000E+10 -1.000000E+10 -1.000000E+10 -1.000000E+10 -1.000000E+10  0.000000E+00  0.000000E+00  0.000000E+00  0.000000E+00  0.000000E+00  0.000000E+00  0.000000E+00  0.000000E+00  0.000000E+00  0.000000E+00  0.000000E+00  0.000000E+00       Funtag_D
      73  5.402452E+05  4.979736E+05       42271.7       5.48495     0.0919677       1.44125      0.209608      0.229702    0.00677470   -0.00106100    0.00806128       1.12394       Opttag_D
      73       46.7860       2.04833       1.00000  0.000000E+00       1.00000  0.000000E+00      0.794868       64.7423       1.00000       25.0733  0.000000E+00      0.963469  0.000000E+00      0.963469  0.000000E+00  0.000000E+00      -1.92694       46.8739       1.38831        1       Scatag_D
      73       1832.15       6894.43  0.000000E+00  0.000000E+00       2283.25  0.000000E+00       3898.52       6160.74  0.000000E+00       11084.0       64.3143       10054.2 -1.000000E+10 -1.000000E+10 -1.000000E+10 -1.000000E+10 -1.000000E+10  0.000000E+00  0.000000E+00  0.000000E+00  0.000000E+00  0.000000E+00  0.000000E+00  0.000000E+00  0.000000E+00  0.000000E+00  0.000000E+00  0.000000E+00  0.000000E+00       Funtag_D
      74  5.402348E+05  4.979759E+05       42258.9       4.64143     0.0922824       1.44970      0.209604      0.229689    0.00676946   -0.00104207    0.00805751       1.12376       Opttag_D
      74       46.7865       2.04856       1.00000  0.000000E+00       1.00000  0.000000E+00      0.794880       64.7444       1.00000       25.0713  0.000000E+00      0.963910  0.000000E+00      0.963910  0.000000E+00  0.000000E+00      -1.92782       46.8739       1.38831        1       Scatag_D
      74       1830.43       6892.33  0.000000E+00  0.000000E+00       2283.36  0.000000E+00       3896.47       6158.87  0.000000E+00       11081.3       64.4069       10051.7 -1.000000E+10 -1.000000E+10 -1.000000E+10 -1.000000E+10 -1.000000E+10  0.000000E+00  0.000000E+00  0.000000E+00  0.000000E+00  0.000000E+00  0.000000E+00  0.000000E+00  0.000000E+00  0.000000E+00  0.000000E+00  0.000000E+00  0.000000E+00       Funtag_D
      75  5.402252E+05  4.979745E+05       42250.7       5.70560     0.0927074       1.46143      0.209596      0.229668    0.00677259 -9.993179E-04    0.00805768       1.12360       Opttag_D
      75       46.7869       2.04870       1.00000  0.000000E+00       1.00000  0.000000E+00      0.794896       64.7462       1.00000       25.0678  0.000000E+00      0.964389  0.000000E+00      0.964389  0.000000E+00  0.000000E+00      -1.92878       46.8739       1.38831        1       Scatag_D
      75       1830.51       6890.30  0.000000E+00  0.000000E+00       2283.49  0.000000E+00       3894.02       6159.50  0.000000E+00       11078.0       64.4501       10050.4 -1.000000E+10 -1.000000E+10 -1.000000E+10 -1.000000E+10 -1.000000E+10  0.000000E+00  0.000000E+00  0.000000E+00  0.000000E+00  0.000000E+00  0.000000E+00  0.000000E+00  0.000000E+00  0.000000E+00  0.000000E+00  0.000000E+00  0.000000E+00       Funtag_D
      76  5.402145E+05  4.979691E+05       42245.4       5.15677     0.0931879       1.47492      0.209589      0.229644    0.00678447 -9.284170E-04    0.00806060       1.12349       Opttag_D
      76       46.7870       2.04873       1.00000  0.000000E+00       1.00000  0.000000E+00      0.794915       64.7465       1.00000       25.0638  0.000000E+00      0.964736  0.000000E+00      0.964736  0.000000E+00  0.000000E+00      -1.92947       46.8739       1.38831        1       Scatag_D
      76       1831.84       6888.94  0.000000E+00  0.000000E+00       2283.64  0.000000E+00       3891.05       6160.44  0.000000E+00       11074.8       64.4768       10050.2 -1.000000E+10 -1.000000E+10 -1.000000E+10 -1.000000E+10 -1.000000E+10  0.000000E+00  0.000000E+00  0.000000E+00  0.000000E+00  0.000000E+00  0.000000E+00  0.000000E+00  0.000000E+00  0.000000E+00  0.000000E+00  0.000000E+00  0.000000E+00       Funtag_D
      77  5.402041E+05  4.979591E+05       42245.0       4.72135     0.0935393       1.48534      0.209577      0.229622    0.00680672 -8.593127E-04    0.00806267       1.12342       Opttag_D
      77       46.7867       2.04854       1.00000  0.000000E+00       1.00000  0.000000E+00      0.794929       64.7452       1.00000       25.0603  0.000000E+00      0.964832  0.000000E+00      0.964832  0.000000E+00  0.000000E+00      -1.92966       46.8739       1.38831        1       Scatag_D
      77       1832.78       6888.17  0.000000E+00  0.000000E+00       2283.74  0.000000E+00       3889.49       6163.56  0.000000E+00       11072.7       64.4701       10050.1 -1.000000E+10 -1.000000E+10 -1.000000E+10 -1.000000E+10 -1.000000E+10  0.000000E+00  0.000000E+00  0.000000E+00  0.000000E+00  0.000000E+00  0.000000E+00  0.000000E+00  0.000000E+00  0.000000E+00  0.000000E+00  0.000000E+00  0.000000E+00       Funtag_D

 An atom has moved more than     1.50 A from the initial/last
 position - if this was an accepted trial point, probably need to update the
 non-bonded contact list.

      78  5.401919E+05  4.979448E+05       42247.1       5.52548     0.0940358       1.50058      0.209563      0.229591    0.00683834 -7.626625E-04    0.00806327       1.12339       Opttag_D
      78       46.7862       2.04826       1.00000  0.000000E+00       1.00000  0.000000E+00      0.794947       64.7446       1.00000       25.0549  0.000000E+00      0.964878  0.000000E+00      0.964878  0.000000E+00  0.000000E+00      -1.92976       46.8739       1.38831        1       Scatag_D
      78       1833.05       6887.70  0.000000E+00  0.000000E+00       2283.95  0.000000E+00       3887.95       6170.35  0.000000E+00       11070.2       64.4842       10049.4 -1.000000E+10 -1.000000E+10 -1.000000E+10 -1.000000E+10 -1.000000E+10  0.000000E+00  0.000000E+00  0.000000E+00  0.000000E+00  0.000000E+00  0.000000E+00  0.000000E+00  0.000000E+00  0.000000E+00  0.000000E+00  0.000000E+00  0.000000E+00       Funtag_D

 An atom has moved more than     1.50 A from the initial/last
 position - need to update the non-bonded contact list.


 ############################################################################################################
 ###################################################### setting up contact terms
 ############################################################################################################

      79  5.401830E+05  4.979329E+05       42250.2       7.56439     0.0946469       1.53680      0.209546      0.229558    0.00686483 -6.703238E-04    0.00806187       1.12340       Opttag_D
      79       46.7856       2.04806       1.00000  0.000000E+00       1.00000  0.000000E+00      0.794965       64.7456       1.00000       25.0482  0.000000E+00      0.964997  0.000000E+00      0.964997  0.000000E+00  0.000000E+00      -1.92999       46.8739       1.38831        1       Scatag_D
      79       1832.42       6887.84  0.000000E+00  0.000000E+00       2284.22  0.000000E+00       3886.07       6178.64  0.000000E+00       11067.9       64.5898       10048.5 -1.000000E+10 -1.000000E+10 -1.000000E+10 -1.000000E+10 -1.000000E+10  0.000000E+00  0.000000E+00  0.000000E+00  0.000000E+00  0.000000E+00  0.000000E+00  0.000000E+00  0.000000E+00  0.000000E+00  0.000000E+00  0.000000E+00  0.000000E+00       Funtag_D
      80  5.401722E+05  4.979313E+05       42240.9       4.94571     0.0947499       1.54144      0.209542      0.229547    0.00686825 -6.494847E-04    0.00805695       1.12335       Opttag_D
      80       46.7853       2.04805       1.00000  0.000000E+00       1.00000  0.000000E+00      0.794965       64.7477       1.00000       25.0457  0.000000E+00      0.965052  0.000000E+00      0.965052  0.000000E+00  0.000000E+00      -1.93010       46.8739       1.38831        1       Scatag_D
      80       1830.18       6887.29  0.000000E+00  0.000000E+00       2284.34  0.000000E+00       3885.69       6175.64  0.000000E+00       11067.4       64.6412       10045.7 -1.000000E+10 -1.000000E+10 -1.000000E+10 -1.000000E+10 -1.000000E+10  0.000000E+00  0.000000E+00  0.000000E+00  0.000000E+00  0.000000E+00  0.000000E+00  0.000000E+00  0.000000E+00  0.000000E+00  0.000000E+00  0.000000E+00  0.000000E+00       Funtag_D
      81  5.401565E+05  4.979303E+05       42226.3       5.45880     0.0951032       1.55675      0.209535      0.229517    0.00687057 -5.975068E-04    0.00804810       1.12328       Opttag_D
      81       46.7848       2.04815       1.00000  0.000000E+00       1.00000  0.000000E+00      0.794966       64.7543       1.00000       25.0390  0.000000E+00      0.965349  0.000000E+00      0.965349  0.000000E+00  0.000000E+00      -1.93070       46.8739       1.38831        1       Scatag_D
      81       1826.16       6886.41  0.000000E+00  0.000000E+00       2284.65  0.000000E+00       3883.74       6174.46  0.000000E+00       11065.8       64.8159       10040.2 -1.000000E+10 -1.000000E+10 -1.000000E+10 -1.000000E+10 -1.000000E+10  0.000000E+00  0.000000E+00  0.000000E+00  0.000000E+00  0.000000E+00  0.000000E+00  0.000000E+00  0.000000E+00  0.000000E+00  0.000000E+00  0.000000E+00  0.000000E+00       Funtag_D
      82  5.401403E+05  4.979260E+05       42214.3       7.52936     0.0957110       1.58266      0.209519      0.229475    0.00688003 -5.092401E-04    0.00804353       1.12324       Opttag_D
      82       46.7838       2.04821       1.00000  0.000000E+00       1.00000  0.000000E+00      0.794971       64.7624       1.00000       25.0289  0.000000E+00      0.965790  0.000000E+00      0.965790  0.000000E+00  0.000000E+00      -1.93158       46.8739       1.38831        1       Scatag_D
      82       1824.09       6885.92  0.000000E+00  0.000000E+00       2285.09  0.000000E+00       3880.64       6174.80  0.000000E+00       11063.7       64.9995       10035.1 -1.000000E+10 -1.000000E+10 -1.000000E+10 -1.000000E+10 -1.000000E+10  0.000000E+00  0.000000E+00  0.000000E+00  0.000000E+00  0.000000E+00  0.000000E+00  0.000000E+00  0.000000E+00  0.000000E+00  0.000000E+00  0.000000E+00  0.000000E+00       Funtag_D
      83  5.401191E+05  4.979170E+05       42202.1       9.51314     0.0970545       1.63018      0.209495      0.229394    0.00689997 -3.303114E-04    0.00804461       1.12331       Opttag_D
      83       46.7814       2.04826       1.00000  0.000000E+00       1.00000  0.000000E+00      0.794979       64.7780       1.00000       25.0091  0.000000E+00      0.966704  0.000000E+00      0.966704  0.000000E+00  0.000000E+00      -1.93341       46.8739       1.38831        1       Scatag_D
      83       1824.58       6886.71  0.000000E+00  0.000000E+00       2285.96  0.000000E+00       3875.05       6174.42  0.000000E+00       11060.5       65.3384       10029.5 -1.000000E+10 -1.000000E+10 -1.000000E+10 -1.000000E+10 -1.000000E+10  0.000000E+00  0.000000E+00  0.000000E+00  0.000000E+00  0.000000E+00  0.000000E+00  0.000000E+00  0.000000E+00  0.000000E+00  0.000000E+00  0.000000E+00  0.000000E+00       Funtag_D
      84  5.400996E+05  4.979081E+05       42191.5       6.96179     0.0974827       1.63792      0.209486      0.229372    0.00691971 -2.759382E-04    0.00804782       1.12322       Opttag_D
      84       46.7803       2.04805       1.00000  0.000000E+00       1.00000  0.000000E+00      0.794978       64.7809       1.00000       25.0029  0.000000E+00      0.966869  0.000000E+00      0.966869  0.000000E+00  0.000000E+00      -1.93374       46.8739       1.38831        1       Scatag_D
      84       1826.03       6885.65  0.000000E+00  0.000000E+00       2286.22  0.000000E+00       3873.99       6168.35  0.000000E+00       11059.3       65.3692       10026.6 -1.000000E+10 -1.000000E+10 -1.000000E+10 -1.000000E+10 -1.000000E+10  0.000000E+00  0.000000E+00  0.000000E+00  0.000000E+00  0.000000E+00  0.000000E+00  0.000000E+00  0.000000E+00  0.000000E+00  0.000000E+00  0.000000E+00  0.000000E+00       Funtag_D
      85  5.400828E+05  4.978960E+05       42186.8       5.60351     0.0976912       1.63522      0.209477      0.229362    0.00694639 -2.560572E-04    0.00805259       1.12315       Opttag_D
      85       46.7792       2.04766       1.00000  0.000000E+00       1.00000  0.000000E+00      0.794972       64.7801       1.00000       24.9996  0.000000E+00      0.966907  0.000000E+00      0.966907  0.000000E+00  0.000000E+00      -1.93381       46.8739       1.38831        1       Scatag_D
      85       1828.20       6884.74  0.000000E+00  0.000000E+00       2286.35  0.000000E+00       3874.76       6165.23  0.000000E+00       11058.5       65.3317       10023.7 -1.000000E+10 -1.000000E+10 -1.000000E+10 -1.000000E+10 -1.000000E+10  0.000000E+00  0.000000E+00  0.000000E+00  0.000000E+00  0.000000E+00  0.000000E+00  0.000000E+00  0.000000E+00  0.000000E+00  0.000000E+00  0.000000E+00  0.000000E+00       Funtag_D
      86  5.400694E+05  4.978819E+05       42187.6       5.63750     0.0980361       1.63526      0.209469      0.229345    0.00697781 -2.303078E-04    0.00805902       1.12314       Opttag_D
      86       46.7778       2.04722       1.00000  0.000000E+00       1.00000  0.000000E+00      0.794967       64.7804       1.00000       24.9949  0.000000E+00      0.966934  0.000000E+00      0.966934  0.000000E+00  0.000000E+00      -1.93387       46.8739       1.38831        1       Scatag_D
      86       1831.12       6884.66  0.000000E+00  0.000000E+00       2286.56  0.000000E+00       3876.75       6164.08  0.000000E+00       11057.8       65.2826       10021.3 -1.000000E+10 -1.000000E+10 -1.000000E+10 -1.000000E+10 -1.000000E+10  0.000000E+00  0.000000E+00  0.000000E+00  0.000000E+00  0.000000E+00  0.000000E+00  0.000000E+00  0.000000E+00  0.000000E+00  0.000000E+00  0.000000E+00  0.000000E+00       Funtag_D
      87  5.400565E+05  4.978635E+05       42192.9       8.55432     0.0987657       1.64405      0.209454      0.229312    0.00701839 -1.954562E-04    0.00806365       1.12317       Opttag_D
      87       46.7757       2.04669       1.00000  0.000000E+00       1.00000  0.000000E+00      0.794966       64.7824       1.00000       24.9857  0.000000E+00      0.967379  0.000000E+00      0.967379  0.000000E+00  0.000000E+00      -1.93476       46.8739       1.38831        1       Scatag_D
      87       1833.23       6885.00  0.000000E+00  0.000000E+00       2286.92  0.000000E+00       3880.59       6165.80  0.000000E+00       11057.2       65.2815       10018.9 -1.000000E+10 -1.000000E+10 -1.000000E+10 -1.000000E+10 -1.000000E+10  0.000000E+00  0.000000E+00  0.000000E+00  0.000000E+00  0.000000E+00  0.000000E+00  0.000000E+00  0.000000E+00  0.000000E+00  0.000000E+00  0.000000E+00  0.000000E+00       Funtag_D
      88  5.400423E+05  4.978574E+05       42184.9       5.43219     0.0990820       1.64936      0.209448      0.229302    0.00703188 -1.998875E-04    0.00806021       1.12314       Opttag_D
      88       46.7749       2.04653       1.00000  0.000000E+00       1.00000  0.000000E+00      0.794964       64.7846       1.00000       24.9817  0.000000E+00      0.967590  0.000000E+00      0.967590  0.000000E+00  0.000000E+00      -1.93518       46.8739       1.38831        1       Scatag_D
      88       1831.66       6884.70  0.000000E+00  0.000000E+00       2287.02  0.000000E+00       3883.01       6159.37  0.000000E+00       11056.9       65.2896       10016.9 -1.000000E+10 -1.000000E+10 -1.000000E+10 -1.000000E+10 -1.000000E+10  0.000000E+00  0.000000E+00  0.000000E+00  0.000000E+00  0.000000E+00  0.000000E+00  0.000000E+00  0.000000E+00  0.000000E+00  0.000000E+00  0.000000E+00  0.000000E+00       Funtag_D
      89  5.400295E+05  4.978533E+05       42176.2       4.78175     0.0995266       1.66057      0.209442      0.229287    0.00704105 -2.057560E-04    0.00805404       1.12313       Opttag_D
      89       46.7740       2.04648       1.00000  0.000000E+00       1.00000  0.000000E+00      0.794964       64.7890       1.00000       24.9760  0.000000E+00      0.967943  0.000000E+00      0.967943  0.000000E+00  0.000000E+00      -1.93589       46.8739       1.38831        1       Scatag_D
      89       1828.86       6884.57  0.000000E+00  0.000000E+00       2287.13  0.000000E+00       3885.16       6154.41  0.000000E+00       11056.5       65.3573       10014.2 -1.000000E+10 -1.000000E+10 -1.000000E+10 -1.000000E+10 -1.000000E+10  0.000000E+00  0.000000E+00  0.000000E+00  0.000000E+00  0.000000E+00  0.000000E+00  0.000000E+00  0.000000E+00  0.000000E+00  0.000000E+00  0.000000E+00  0.000000E+00       Funtag_D
      90  5.400188E+05  4.978464E+05       42172.4       5.58948      0.100099       1.67481      0.209433      0.229269    0.00705635 -2.061153E-04    0.00805327       1.12323       Opttag_D
      90       46.7728       2.04635       1.00000  0.000000E+00       1.00000  0.000000E+00      0.794962       64.7944       1.00000       24.9686  0.000000E+00      0.968456  0.000000E+00      0.968456  0.000000E+00  0.000000E+00      -1.93691       46.8739       1.38831        1       Scatag_D
      90       1828.51       6885.74  0.000000E+00  0.000000E+00       2287.25  0.000000E+00       3886.84       6150.11  0.000000E+00       11056.1       65.4428       10012.4 -1.000000E+10 -1.000000E+10 -1.000000E+10 -1.000000E+10 -1.000000E+10  0.000000E+00  0.000000E+00  0.000000E+00  0.000000E+00  0.000000E+00  0.000000E+00  0.000000E+00  0.000000E+00  0.000000E+00  0.000000E+00  0.000000E+00  0.000000E+00       Funtag_D
      91  5.400077E+05  4.978378E+05       42169.9       6.12008      0.100932       1.69933      0.209422      0.229246    0.00707539 -2.106664E-04    0.00805703       1.12346       Opttag_D
      91       46.7710       2.04620       1.00000  0.000000E+00       1.00000  0.000000E+00      0.794957       64.8027       1.00000       24.9583  0.000000E+00      0.969072  0.000000E+00      0.969072  0.000000E+00  0.000000E+00      -1.93814       46.8739       1.38831        1       Scatag_D
      91       1830.22       6888.54  0.000000E+00  0.000000E+00       2287.38  0.000000E+00       3888.53       6142.71  0.000000E+00       11055.9       65.5956       10011.0 -1.000000E+10 -1.000000E+10 -1.000000E+10 -1.000000E+10 -1.000000E+10  0.000000E+00  0.000000E+00  0.000000E+00  0.000000E+00  0.000000E+00  0.000000E+00  0.000000E+00  0.000000E+00  0.000000E+00  0.000000E+00  0.000000E+00  0.000000E+00       Funtag_D
      92  5.399977E+05  4.978296E+05       42168.2       4.89435      0.101281       1.71068      0.209415      0.229241    0.00709361 -2.068339E-04    0.00806241       1.12350       Opttag_D
      92       46.7701       2.04598       1.00000  0.000000E+00       1.00000  0.000000E+00      0.794956       64.8047       1.00000       24.9545  0.000000E+00      0.969192  0.000000E+00      0.969192  0.000000E+00  0.000000E+00      -1.93838       46.8739       1.38831        1       Scatag_D
      92       1832.66       6889.12  0.000000E+00  0.000000E+00       2287.44  0.000000E+00       3888.73       6139.31  0.000000E+00       11056.0       65.6295       10009.4 -1.000000E+10 -1.000000E+10 -1.000000E+10 -1.000000E+10 -1.000000E+10  0.000000E+00  0.000000E+00  0.000000E+00  0.000000E+00  0.000000E+00  0.000000E+00  0.000000E+00  0.000000E+00  0.000000E+00  0.000000E+00  0.000000E+00  0.000000E+00       Funtag_D
      93  5.399860E+05  4.978155E+05       42170.5       4.98928      0.101767       1.72846      0.209403      0.229234    0.00712483 -1.965341E-04    0.00807106       1.12359       Opttag_D
      93       46.7689       2.04558       1.00000  0.000000E+00       1.00000  0.000000E+00      0.794955       64.8065       1.00000       24.9498  0.000000E+00      0.969250  0.000000E+00      0.969250  0.000000E+00  0.000000E+00      -1.93850       46.8739       1.38831        1       Scatag_D
      93       1836.60       6890.21  0.000000E+00  0.000000E+00       2287.51  0.000000E+00       3889.59       6137.47  0.000000E+00       11056.4       65.6391       10007.1 -1.000000E+10 -1.000000E+10 -1.000000E+10 -1.000000E+10 -1.000000E+10  0.000000E+00  0.000000E+00  0.000000E+00  0.000000E+00  0.000000E+00  0.000000E+00  0.000000E+00  0.000000E+00  0.000000E+00  0.000000E+00  0.000000E+00  0.000000E+00       Funtag_D
      94  5.399739E+05  4.977950E+05       42178.9       6.00452      0.102588       1.75975      0.209390      0.229222    0.00717024 -1.812042E-04    0.00808079       1.12380       Opttag_D
      94       46.7672       2.04503       1.00000  0.000000E+00       1.00000  0.000000E+00      0.794960       64.8095       1.00000       24.9426  0.000000E+00      0.969293  0.000000E+00      0.969293  0.000000E+00  0.000000E+00      -1.93859       46.8739       1.38831        1       Scatag_D
      94       1841.03       6892.75  0.000000E+00  0.000000E+00       2287.65  0.000000E+00       3892.66       6136.15  0.000000E+00       11057.8       65.6103       10005.3 -1.000000E+10 -1.000000E+10 -1.000000E+10 -1.000000E+10 -1.000000E+10  0.000000E+00  0.000000E+00  0.000000E+00  0.000000E+00  0.000000E+00  0.000000E+00  0.000000E+00  0.000000E+00  0.000000E+00  0.000000E+00  0.000000E+00  0.000000E+00       Funtag_D
      95  5.399627E+05  4.977678E+05       42194.9       7.00353      0.103712       1.80306      0.209370      0.229204    0.00723056 -1.418016E-04    0.00808917       1.12401       Opttag_D
      95       46.7652       2.04433       1.00000  0.000000E+00       1.00000  0.000000E+00      0.794972       64.8134       1.00000       24.9328  0.000000E+00      0.969282  0.000000E+00      0.969282  0.000000E+00  0.000000E+00      -1.93856       46.8739       1.38831        1       Scatag_D
      95       1844.85       6895.31  0.000000E+00  0.000000E+00       2287.77  0.000000E+00       3898.25       6137.55  0.000000E+00       11060.7       65.5408       10004.9 -1.000000E+10 -1.000000E+10 -1.000000E+10 -1.000000E+10 -1.000000E+10  0.000000E+00  0.000000E+00  0.000000E+00  0.000000E+00  0.000000E+00  0.000000E+00  0.000000E+00  0.000000E+00  0.000000E+00  0.000000E+00  0.000000E+00  0.000000E+00       Funtag_D
      96  5.399520E+05  4.977588E+05       42193.2       4.61884      0.104113       1.81894      0.209362      0.229194    0.00725040 -1.295856E-04    0.00808375       1.12403       Opttag_D
      96       46.7648       2.04413       1.00000  0.000000E+00       1.00000  0.000000E+00      0.794980       64.8149       1.00000       24.9292  0.000000E+00      0.969431  0.000000E+00      0.969431  0.000000E+00  0.000000E+00      -1.93886       46.8739       1.38831        1       Scatag_D
      96       1842.38       6895.60  0.000000E+00  0.000000E+00       2287.76  0.000000E+00       3900.67       6136.01  0.000000E+00       11061.6       65.5124       10003.6 -1.000000E+10 -1.000000E+10 -1.000000E+10 -1.000000E+10 -1.000000E+10  0.000000E+00  0.000000E+00  0.000000E+00  0.000000E+00  0.000000E+00  0.000000E+00  0.000000E+00  0.000000E+00  0.000000E+00  0.000000E+00  0.000000E+00  0.000000E+00       Funtag_D
      97  5.399422E+05  4.977479E+05       42194.3       4.16624      0.104611       1.83823      0.209352      0.229180    0.00727457 -1.112615E-04    0.00807644       1.12411       Opttag_D
      97       46.7645       2.04394       1.00000  0.000000E+00       1.00000  0.000000E+00      0.794992       64.8179       1.00000       24.9242  0.000000E+00      0.969602  0.000000E+00      0.969602  0.000000E+00  0.000000E+00      -1.93920       46.8739       1.38831        1       Scatag_D
      97       1839.04       6896.56  0.000000E+00  0.000000E+00       2287.70  0.000000E+00       3903.60       6137.10  0.000000E+00       11062.6       65.5111       10002.1 -1.000000E+10 -1.000000E+10 -1.000000E+10 -1.000000E+10 -1.000000E+10  0.000000E+00  0.000000E+00  0.000000E+00  0.000000E+00  0.000000E+00  0.000000E+00  0.000000E+00  0.000000E+00  0.000000E+00  0.000000E+00  0.000000E+00  0.000000E+00       Funtag_D
      98  5.399341E+05  4.977312E+05       42202.9       4.97331      0.105272       1.86224      0.209336      0.229164    0.00731142 -8.060173E-05    0.00807529       1.12428       Opttag_D
      98       46.7640       2.04360       1.00000  0.000000E+00       1.00000  0.000000E+00      0.795005       64.8210       1.00000       24.9175  0.000000E+00      0.969879  0.000000E+00      0.969879  0.000000E+00  0.000000E+00      -1.93976       46.8739       1.38831        1       Scatag_D
      98       1838.52       6898.71  0.000000E+00  0.000000E+00       2287.61  0.000000E+00       3906.49       6140.27  0.000000E+00       11063.8       65.5608       10002.0 -1.000000E+10 -1.000000E+10 -1.000000E+10 -1.000000E+10 -1.000000E+10  0.000000E+00  0.000000E+00  0.000000E+00  0.000000E+00  0.000000E+00  0.000000E+00  0.000000E+00  0.000000E+00  0.000000E+00  0.000000E+00  0.000000E+00  0.000000E+00       Funtag_D
      99  5.399223E+05  4.977027E+05       42219.6       4.97778      0.106382       1.90165      0.209311      0.229142    0.00737456 -3.856428E-05    0.00807654       1.12467       Opttag_D
      99       46.7632       2.04302       1.00000  0.000000E+00       1.00000  0.000000E+00      0.795027       64.8267       1.00000       24.9066  0.000000E+00      0.970283  0.000000E+00      0.970283  0.000000E+00  0.000000E+00      -1.94057       46.8739       1.38831        1       Scatag_D
      99       1839.09       6903.46  0.000000E+00  0.000000E+00       2287.40  0.000000E+00       3910.41       6144.89  0.000000E+00       11065.5       65.7347       10003.1 -1.000000E+10 -1.000000E+10 -1.000000E+10 -1.000000E+10 -1.000000E+10  0.000000E+00  0.000000E+00  0.000000E+00  0.000000E+00  0.000000E+00  0.000000E+00  0.000000E+00  0.000000E+00  0.000000E+00  0.000000E+00  0.000000E+00  0.000000E+00       Funtag_D

   MaxLik Scaling Cycle 0105

   Wilson plot

   Statistics in resolution bins after refinement cycle 0003
```


|  |  |
| --- | --- |
|  | - Rxpct factors plot- Rxpct factors table- <Log-Lik Gain>- Ln Sigma A- Correlation Coefficients |


```
   Iters     Tot_Funct    Xray_Funct    Geom_Funct          Grms     rmsD2init     maxD2init             R         Rfree           LLG       LLGfree     rms(bond)    rms(angle)
   Iters             K   B_IMPF_FRAG        K_MISS   B_TEMP_MISS   K_IMPF_MISS   B_IMPF_MISS        K_SOLV   B_TEMP_SOLV   K_IMPF_SOLV   B_IMPF_SOLV        B_TEMP     B_TEMP_11     B_TEMP_12     B_TEMP_22     B_TEMP_13     B_TEMP_23     B_TEMP_33      K_WILSON      B_WILSON      FGCYCLES
   Iters          BOND         ANGLE      IMPROPER       TORSION    SINTORSION      TRIGONAL        PLANAR  BCORRELATION       CONTACT     IDEALDIST        CHIRAL    SIMILARITY       OCCSSUM  UTILDISTANCE     UTILANGLE   UTILTORSION        RHOFIT        ADP-01        ADP-02        ADP-03        ADP-04        ADP-05        ADP-06        ADP-07        ADP-08        ADP-09        ADP-10      RHOFITPC            QM
     100  5.399110E+05  4.976777E+05       42233.2       5.80020      0.107333       1.93316      0.209289      0.229129    0.00742995  1.089860E-05    0.00808204       1.12493       Opttag_D
     100       46.7625       2.04246       1.00000  0.000000E+00       1.00000  0.000000E+00      0.795047       64.8297       1.00000       24.8984  0.000000E+00      0.970529  0.000000E+00      0.970529  0.000000E+00  0.000000E+00      -1.94106       46.8739       1.38831        1       Scatag_D
     100       1841.60       6906.64  0.000000E+00  0.000000E+00       2287.18  0.000000E+00       3912.23       6149.04  0.000000E+00       11066.5       65.9421       10004.1 -1.000000E+10 -1.000000E+10 -1.000000E+10 -1.000000E+10 -1.000000E+10  0.000000E+00  0.000000E+00  0.000000E+00  0.000000E+00  0.000000E+00  0.000000E+00  0.000000E+00  0.000000E+00  0.000000E+00  0.000000E+00  0.000000E+00  0.000000E+00       Funtag_D
   Geometry Restraint Deviations SCREEN Information

 
 No -bcorrel_zeroocchydrogens_factor flag found - keeping default: will set B for OCC=0 hydrogens to    1.200 times heavy-atom B


 TLS information. Total number of groups:     2


 Group   Natoms    FixRB   FixTLS   Group Tag    Group Spec
     1     2785      Y        Y     tls1    { A|* }
     2     2772      Y        Y     tls2    { B|* }
 
NOTE BUSTER_TLS_SET tls1 { A|* }
NOTE BUSTER_TLS_T   tls1     -1.42739E-01 -2.31034E-02  1.80298E-01 -3.76226E-02  2.26092E-02  1.74795E-02
NOTE BUSTER_TLS_L   tls1      1.45453E+00  1.61782E+00  8.45083E-01  9.10505E-02 -6.05438E-01  5.77972E-01
NOTE BUSTER_TLS_S   tls1      2.23750E-01 -2.44115E-01  3.38595E-01 -6.35630E-02  3.30303E-03 -2.70940E-01 -8.61559E-02 -8.56052E-02
NOTE BUSTER_TLS_O   tls1     -2.33111E+01 -8.37601E+01  1.13266E+01
NOTE BUSTER_TLS_FIX tls1 ALL
 
NOTE BUSTER_TLS_SET tls2 { B|* }
NOTE BUSTER_TLS_T   tls2     -1.96623E-01 -1.35074E-01  3.50236E-01  1.18743E-02  4.75488E-02 -1.48386E-02
NOTE BUSTER_TLS_L   tls2      1.48927E+00  9.09257E-01  1.09932E+00  7.78817E-01 -9.29773E-01 -7.15920E-01
NOTE BUSTER_TLS_S   tls2     -3.97061E-02  1.25383E-01 -1.13212E-01  1.50247E-01  1.99842E-01  3.65144E-02 -6.77258E-02  2.74050E-02
NOTE BUSTER_TLS_O   tls2      2.21121E+01 -7.20987E+01  3.46915E+00
NOTE BUSTER_TLS_FIX tls2 ALL

 For final position, Geometry function summary
     term                           number     weight       rms        GooF      function     fn/numb
     ================================================================================================
     BOND bond lengths (angs)       5676       2.000      0.0081       0.403      1841.60       0.324
     ANGL bond angles (degs)        7707       2.000        1.12       0.669      6906.64       0.896
     TORS split-harmonic tors       1281       0.000       17.39       1.091         0.00       0.000
     SINTOR sinusoidal tors         1281       2.000       17.39                  2287.18       1.785
     PLAN general plane (angs)       966       5.000      0.0180       0.900      3912.23       4.050
     BCORrelations (angs^2)         5676      10.000       0.922       0.329      6149.04       1.083
     CONT (bad contacts)               0       5.000                                 0.00
     IDEAL(ideal-dist contact)      5083       4.000                             11066.49
     CHIRAL (gelly semiharmon)       740       5.000                                65.94
     SIM similarity restraint for NCS and/or TARGET                              10004.11
     OCCSUM restraint on sum of occupanies                                           0.00       as none defined
     DISTAN utility restraints interatomic distance                                  0.00       as none defined
     UTILANGLE utility angle restraints                                              0.00       as none defined
     UTILTOR utility restraints on torsion angles                                    0.00       as none defined
     Peptide omega torsion angles                           3.38 degs - monitor only
 
 WARNING: Have |delta/sigma| deviations > 5.0 sigma. Number of outliers for each term: 
 WARNING:         6 bond angles.  Worst is    19.1 sigs    62.80 degs A|501:C10=C05=C04 (LIG)
 WARNING:         1 planes.       Worst is    12.2 sigs     0.24 Angs A|501:N03=C04=C05=C06=C07=C08=C09=C10 (LIG)
 WARNING:         1 idealD contacts. Worst     7.4 sigs     2.18 Angs A|501:C09=C11 (LIG)

 Weighted rms      0.0081         1.125  (temporary rms bond and angle for BUSTER to pick up!)
 Weighted intra-residual rms(bond) for StdProtein (excluding H)      0.0077
 Weighted intra-residual rms(bond) for Buffer     (excluding H)      0.0031
 Outputing geometry progress graph in plotmtv format to file:Geom_Res.mtv

 ############### Running final geometry/screen analysis (screen_final.txt)

 s/r gelly normal termination
```

Statistics and scale factors vs. cycle number
```

|  |  |  |
| --- | --- | --- |
|  | - Rxpct factors- <Log-Lik Gain>- GEOM Residuals- Ln Sigma_A_ | - Scale Factors- B Factors- Imperfection Factors |

```
 Writing F_calc(model+solvent) to complete.mtz

   Final Geometry Information


---


### 2. Final results

Statistics and scale factors vs. cycle number
```

|  |  |  |
| --- | --- | --- |
|  | - Rxpct factors- <Log-Lik Gain>- GEOM Residuals- Ln Sigma_A_ | - Scale Factors- B Factors- Imperfection Factors |

```
   Average Figure of Merit
   Structure Factor Amplitudes


 # normal termination

  Program stopped.
```

closesok message: time spent in tree = 1797.20 (user) 39.28 (sys)

---

# Normal termination

```
 Resources usage summary:
     Time spent in client ...................  1797.21 (user)       39.30 (system)
     Time spent in scripts (    27 calls) ...     3.08 (user)        0.19 (system)
     Time spent in server ...................     0.00 (user)        0.01 (system)

     Max resident memory used (kb) .......... 477292
     Number of page faults .................. 0 (major)    1625760 (minor)
     Number of context switches ............. 12050 (niced)    6825 (other)
```
